# Pd_2_Spermine as an Alternative Therapeutics for Cisplatin-Resistant Triple-Negative Breast Cancer

**DOI:** 10.1021/acs.jmedchem.4c00435

**Published:** 2024-04-09

**Authors:** Tatiana J. Carneiro, Ana L. M. Batista de Carvalho, Martin Vojtek, Raquel C. Laginha, Maria Paula M. Marques, Carmen Diniz, Ana M. Gil

**Affiliations:** † Department of Chemistry and CICECO − Aveiro Institute of Materials, University of Aveiro, 3810-193 Aveiro, Portugal; ‡ Molecular Physical-Chemistry R&D Unit, Department of Chemistry, University of Coimbra, 3004-535 Coimbra, Portugal; § LAQV/REQUIMTE, Laboratory of Pharmacology, Department of Drug Sciences, Faculty of Pharmacy, University of Porto, 4150-755 Porto, Portugal; ∥ Department of Life Sciences, Faculty of Science and Technology, University of Coimbra, 3000-456 Coimbra, Portugal

## Abstract

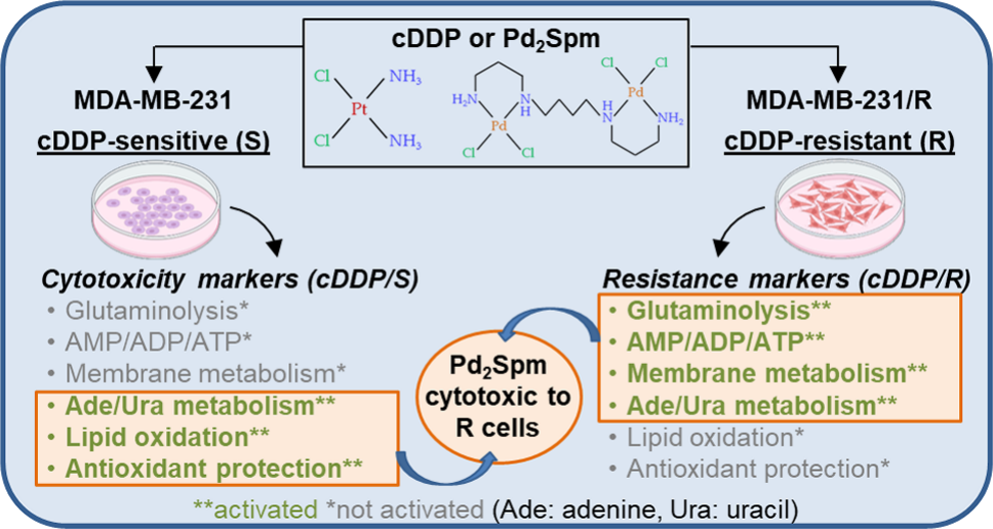

Cisplatin (cDDP) resistance is a matter of concern in triple-negative breast cancer therapeutics. We measured the metabolic response of cDDP-sensitive (S) and -resistant (R) MDA-MB-231 cells to Pd_2_Spermine(Spm) (a possible alternative to cDDP) compared to cDDP to investigate (i) intrinsic response/resistance mechanisms and (ii) the potential cytotoxic role of Pd_2_Spm. Cell extracts were analyzed by untargeted nuclear magnetic resonance metabolomics, and cell media were analyzed for particular metabolites. CDDP-exposed S cells experienced enhanced antioxidant protection and small deviations in the tricarboxylic acid cycle (TCA), pyrimidine metabolism, and lipid oxidation (proposed cytotoxicity signature). R cells responded more strongly to cDDP, suggesting a resistance signature of activated TCA cycle, altered AMP/ADP/ATP and adenine/uracil fingerprints, and phospholipid biosynthesis (without significant antioxidant protection). Pd_2_Spm impacted more markedly on R/S cell metabolisms, inducing similarities to cDDP/S cells (probably reflecting high cytotoxicity) and strong additional effects indicative of amino acid depletion, membrane degradation, energy/nucleotide adaptations, and a possible beneficial intracellular γ-aminobutyrate/glutathione-mediated antioxidant mechanism.

## Introduction

Triple-negative breast cancer (TNBC) is a subtype of breast cancer (BC) that affects approximately 10–20% of patients and is characterized by the absence of hormone receptors (both estrogen and progesterone) and of human epidermal growth factor receptor 2.^1,2^ TNBC is characterized by high metastatic potential and risk of relapse, thus leading to poor prognosis, usually associated with limited treatment options.^3,4^ First-line treatments are usually based on anthracycline and taxane, whereas cytotoxic platinum(II) [Pt(II)] agents (in particular, cisplatin, cDDP) are often used in neoadjuvant therapy, increasing positive responses from about 30% up to 50%.^5^ However, the efficacy of Pt(II) drugs is often hampered by toxicity and acquired resistance.^6,7^ The latter (defined as tumor relapse within 6 months of initial treatment) is particularly critical in TNBC where intrinsic tumor heterogeneity is associated with increased resistance and relapse, compared to other BC subtypes.^8^ These drawbacks of Pt(II)-based therapy have motivated the search for other metal-based drugs, including palladium [Pd(II)] complexes.^9−11^ In particular, the Pd(II) dinuclear chelate with the biogenic polyamine spermine Pd_2_Spm (Spm, H_2_N(CH_2_)_3_NH(CH_2_)_4_NH(CH_2_)_3_NH_2_) exhibited promising *in vitro* antiproliferative, antimigratory, and antiangiogenic properties against the TNBC MDA-MB-231 cell line, along with effective responses in other cancer cell lines, e.g., leukemia, osteosarcoma, oral squamous cells, ovarian, and prostate carcinomas.^12−19^ In addition, *in vivo* studies have shown a more favorable biodistribution profile of Pd(II) in healthy BALB/c mice compared to that of Pt(II), while exposure of a MDA-MB-231 cell-derived xenograft (CDX) mouse model to Pd_2_Spm resulted in the reduction of tumor size and cell proliferation rate, as well as lower systemic toxicity, compared to cDDP.^20,21^ Pd_2_Spm also appears to be more selective for TNBC cells, having less deleterious effects on noncancerous breast cells, at least viewed under *in vitro* conditions.^21^ Metabolomics has been extensively highlighted as a valuable tool towards the understanding of the interplay between drugs and cellular metabolism in breast cancer.^22^ Indeed, metabolomics of CDX mice with TNBC showed that, compared to cDDP, Pd_2_Spm induced (i) pronounced metabolic disturbances in tumor metabolism (energy, membrane, nucleotides, and one-carbon metabolisms), possibly reflecting a different mechanism of action of the Pd(II) complex, and (ii) an enhanced neuroprotective response of brain, along with lower impact on liver.^23,24^ Furthermore, Pd_2_Spm administration induced initial metabolic deviations in healthy BALB/c mice, which, however, returned to control levels faster (within 48 h) than with cDDP, namely, in kidney, liver, breast tissue and brain.^25,26^ This corroborated other reports of lower *in vivo* toxicity of the Pd(II) compound, compared to that of cDDP.^20,21^

In an attempt to address cDDP resistance, the biochemical effects of Pd(II) agents on cDDP-sensitive and cDDP-resistant MDA-MB-231 cells have been investigated.^27^ The trinuclear chelate with spermidine (Spd, H_2_N(CH_2_)_3_NH(CH_2_)_4_NH_2_), Pd_3_Sdp_2_, was compared with its Pt(II)-analog Pt_3_Spd_2_, Pd_2_Spm and cDDP. The Pd(II) complexes exhibited similar enhanced antiproliferative effects in resistant cells compared to cDDP, whereas cDDP remained the most cytotoxic agent for sensitive cells (IC_50_ after 48 h: cDDP 1 μM; Pd_2_Spm 7.90 μM; Pd_3_Spd_2_ 8.44 μM).^27^ The authors suggested that the Pd(II) complexes may share a similar and effective mechanism of action against cDDP-resistant TNBC cells, probably involving other pharmacological targets besides DNA (e.g., proteins or even intracellular water).

The aim of this work was to investigate the effects of Pd_2_Spm and cDDP on the metabolic profiles of cDDP-resistant (R) and cDDP-sensitive (S) MDA-MB-231 cells, measured by nuclear magnetic resonance (NMR) metabolomics. This builds upon a recently reported metabolic signature of untreated cDDP-resistant cells compared to that of sensitive cells,^28^ helping to identify metabolic markers descriptive of the mechanisms of (i) resistance or sensitivity to cDDP and of (ii) the impact of Pd_2_Spm, to investigate its potential to alter/treat cDDP resistance.

## Results

### NMR Spectra of Treated cDDP-Resistant (R) and -Sensitive (S) Cells Compared to Those of Untreated Cells

**Figure 1:**
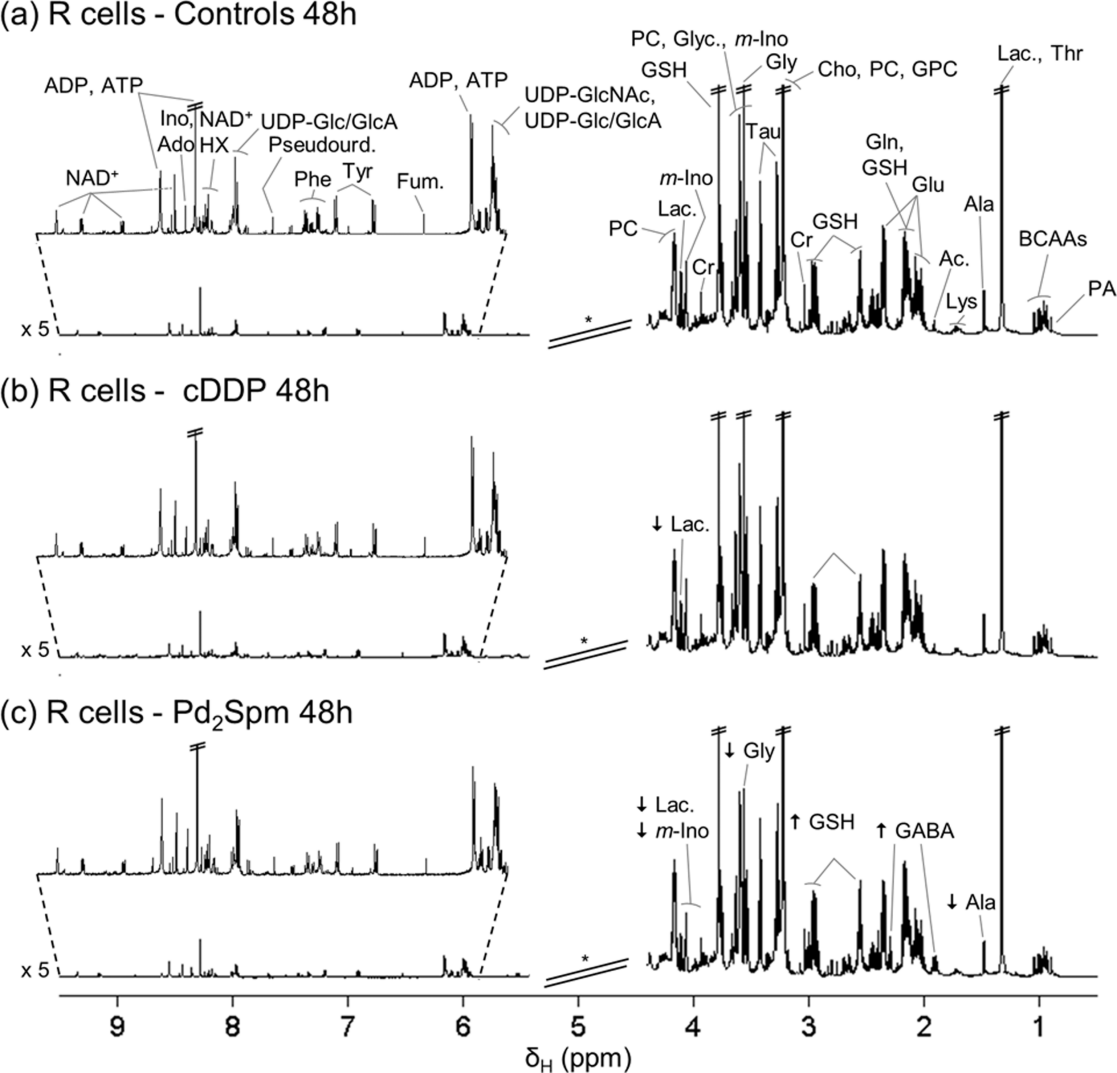
Average ^1^H-NMR spectra of aqueous extracts of cDDP-resistant MDA-MB-231/R cells (48 h) (a) untreated and treated with (b) cDDP and (c) Pd_2_Spm. * Cutoff of water suppression region (δ 4.4–5.4), not considered in the multivariate analysis. The arrows identify metabolic variations found with the visual inspection of spectra of treated groups in relation to controls. Abbreviations: 3-letter code for amino acids; Ac., acetate; Ado, adenosine; ADP, adenosine diphosphate; ATP, adenosine triphosphate; BCAAs, branched-chain amino acids (Ile, Leu, and Val); Cho, choline; Cr, creatine; Fum., fumarate; GABA, γ-aminobutyrate; Glyc., glycerol; GPC, glycerophosphocholine; GSH, glutathione (reduced); HX, hypoxanthine; Ino, inosine; *m*-Ino, *myo*-inositol; Lac., lactate; NAD^+^, nicotinamide adenine dinucleotide (oxidized); PA, pantothenate; PC, phosphocholine; Pseudourd., pseudouridine; Tau, taurine; UDP-Glc/GlcA, uridine diphosphate-glucose/glucuronate; UDP-GlcNAc, uridine diphosphate-*N*-acetylglucosamine.

The ^1^H-NMR spectra of control (untreated) polar extracts of R cells and S cells (at 48 h) are shown in Figures 1a and S1a, respectively. Considering the spectra of R cell extracts (Figure 1), an apparent decrease is noted in lactate upon exposure to cDDP (Figure 1b) compared to controls (Figure 1a). Exposure to Pd_2_Spm also seems to induce a decrease in lactate levels, although other relevant changes include increases in glutathione (GSH) and γ-aminobutyrate (GABA), among other changes (Figure 1c).

On the other hand, the NMR spectra of S cells show no clear change in lactate upon cDDP exposure (Figure S1b), although a decrease in this metabolite is again observed upon Pd_2_Spm treatment (Figure S1c), together with increased GSH and GABA. Furthermore, both metal compounds appear to induce different profiles regarding amino acids and nucleotides in each cell line (Figures 1 and S1 for R and S cell lines, respectively), the details of which will be further explored through statistical analysis.

### Metabolic Profiling of cDDP Impact on cDDP-Resistant (R) and -Sensitive (S) Cells

**Figure 2:**
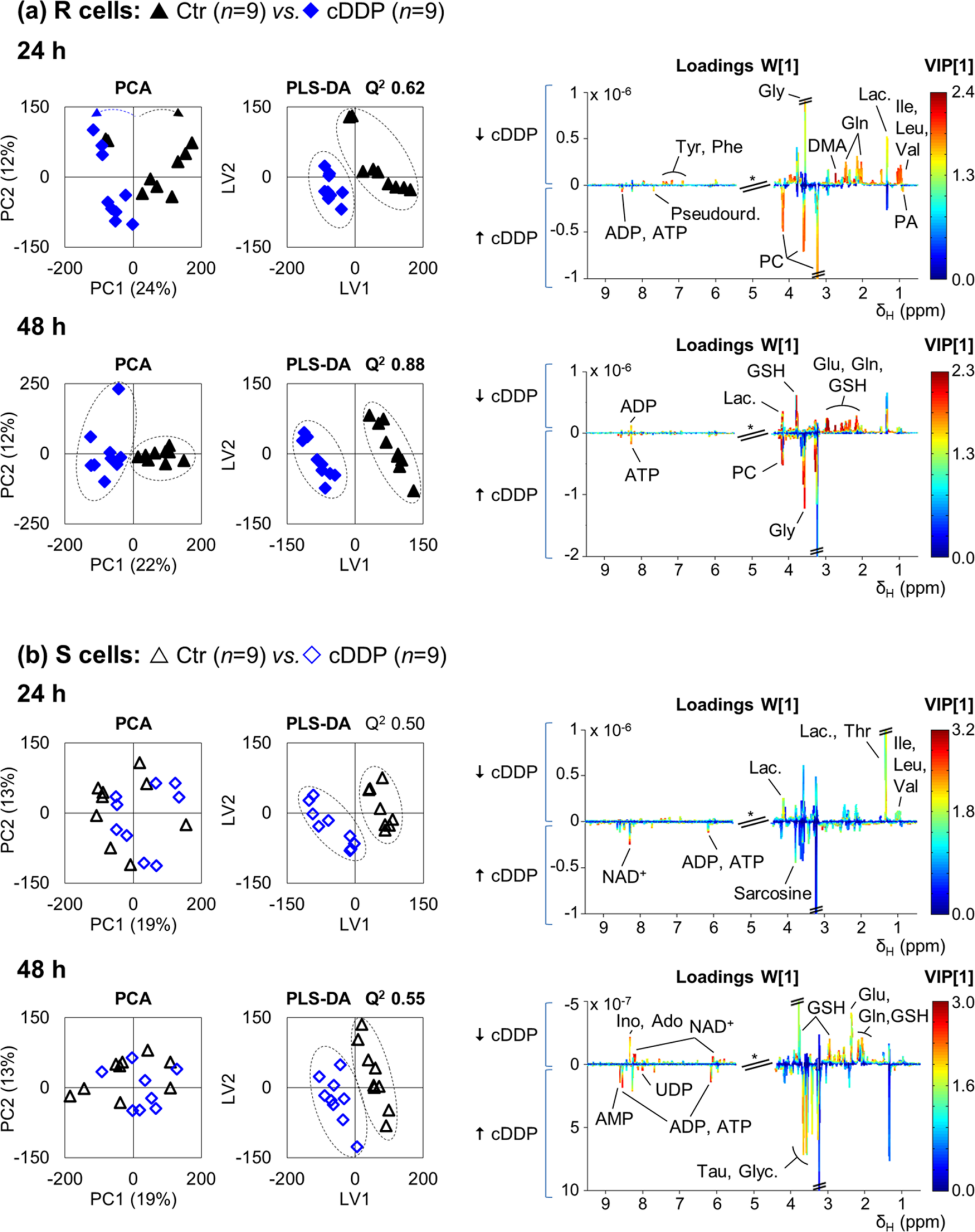
Pairwise PCA and PLS-DA scores and PLS-DA loading plots for ^1^H-NMR spectra of aqueous extracts of cDDP-treated cells. (a) MDA-MB-231/R (R) and (b) MDA-MB-231 (S) cells. CDDP-treated samples (blue diamonds, *n* = 9) vs controls (black triangles, *n* = 9) at 24 and 48 h after treatment. Validation parameters (*R*^2^ and *Q*^2^) are shown for the PLS-DA model. Loading peak assignments are indicated for the most relevant metabolites according to VIP. Abbreviations: AMP, adenosine monophosphate; DMA, dimethylamine; UDP, uridine diphosphate; other abbreviations as defined in the caption of Figure 1.

PCA shows a clearer separation of controls (untreated) in cDDP-exposed R cells (Figure 2a, left) compared to S cells (Figure 2b, left). This suggests that cDDP has a stronger effect on the metabolism of R cells than on that of S cells. This observation refutes intuitive expectations that cDDP resistance would correspond to higher metabolic stability and, instead, suggests that a stronger response may reflect a possible adaptative behavior of R cell metabolism to cDDP. This result is indeed confirmed by the higher predictive power (*Q*^2^) of the PLS-DA models obtained for R cells (*Q*^2^ 0.62 (24 h) and 0.88 (48 h); Figure 2a), compared to those obtained for S cells (*Q*^2^ 0.5 (24 h) and 0.55 (48 h); Figure 2b), as well as by the red-dominated loading plots obtained for R cells (suggestive of larger variation; Figure 2a, right).

**Figure 3:**
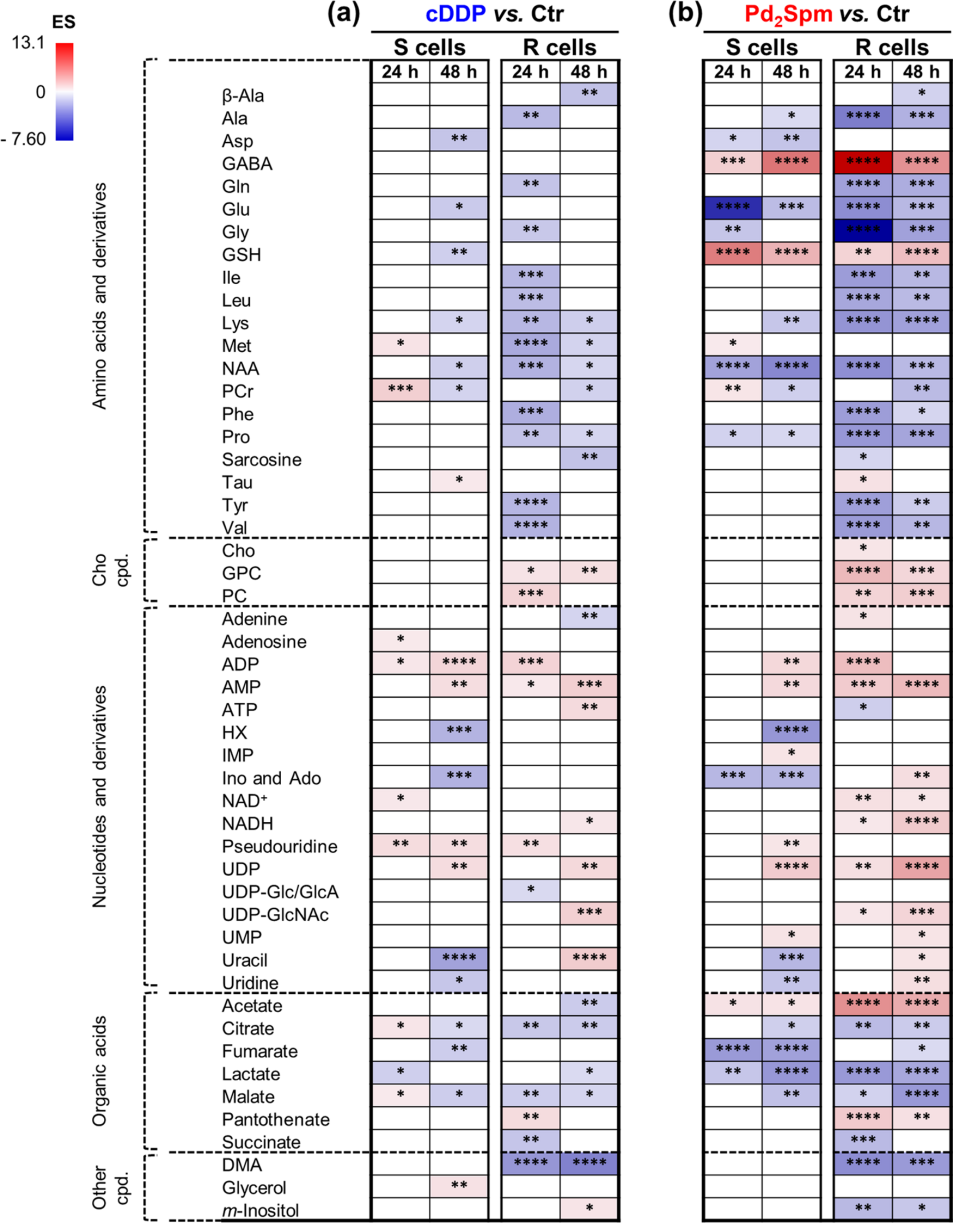
Heatmap of significant effect size variations for (a) cDDP-treated and (b) Pd_2_Spm-treated MDA-MB-231 (S) and MDA-MB-231/R (R) cells compared to controls (untreated cells). Increasing values of effect size (ES) are colored from blue to red corresponding to negative and positive values, respectively, and are not correlated with the generic color used to designate each drug. Abbreviations: IMP, inosine monophosphate; NAA, *N*-acetyl-aspartate; NADH, nicotinamide adenine dinucleotide (reduced); PCr, phosphocreatine; UMP, uridine monophosphate; other abbreviations as defined in the captions of Figures 1 and 2. *p-value <0.05; **p-value <0.01; ***p-value <0.001; ****p-value <0.0001 for the comparison drug *vs* controls at each time-point.

Indeed, clearly distinct patterns of variation are found for cDDP-exposed R cells (vs controls) compared to those for S cells (Figure 3 and Table S1). Concerning amino acids, R cells respond with an early (24 h) depletion of most amino acids, while only methionine and phosphocreatine (PCr) vary (increase) in S cells. Subsequently (48 h), many amino acids recover to control levels in R cells (blank rectangles) or to less significant decreases compared to controls. In spite of the final (48 h) amino acid pattern of R cells (relative to controls) sharing some features in common with that in S cells (decreased lysine, *N*-acetyl-aspartate (NAA) and PCr), it also shows clear distinctions: (i) persistent decreases in β-alanine, methionine, proline, and sarcosine (none changed in S cells at 48 h) and (ii) no increase in taurine nor decreases in aspartate, glutamate, or GSH (contrary to that observed in S cells).

**Figure 4:**
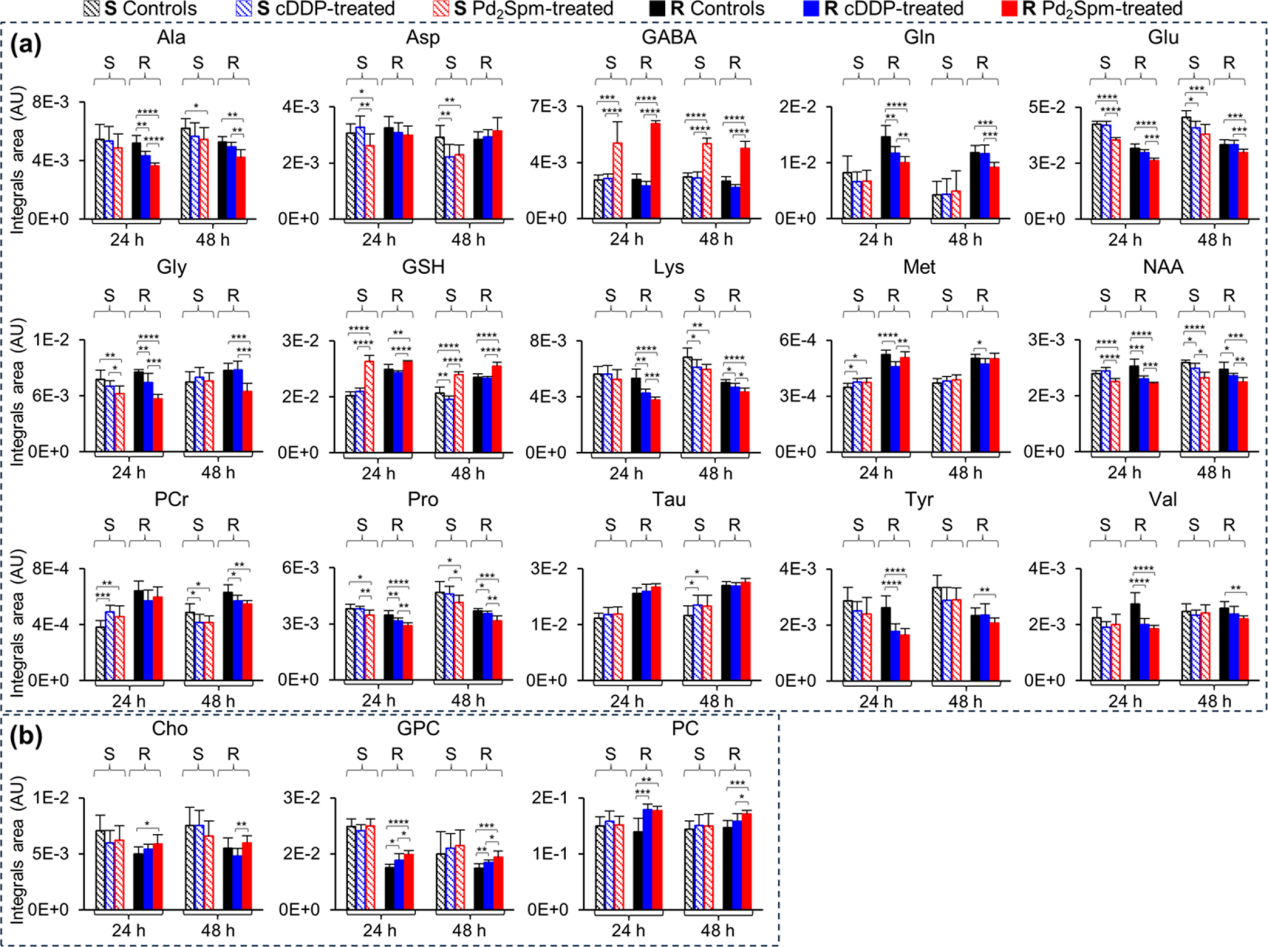
Time-course variations of selected (a) amino acids and (b) choline compounds in MDA-MB-231 (S, striped bars) and MDA-MB-231/R (R, full bars) cells, treated with PBS (controls, black), cDDP (blue), or Pd_2_Spm (red). Values are expressed as the means of normalized peak areas ± SEM. Abbreviations are defined in the captions of Figures 1 and 3. Significant differences between treated groups (R and S cells) and controls, at each time-point: *p-value <0.05; **p-value <0.01; ***p-value <0.001; ****p-value <0.0001.

**Figure 5:**
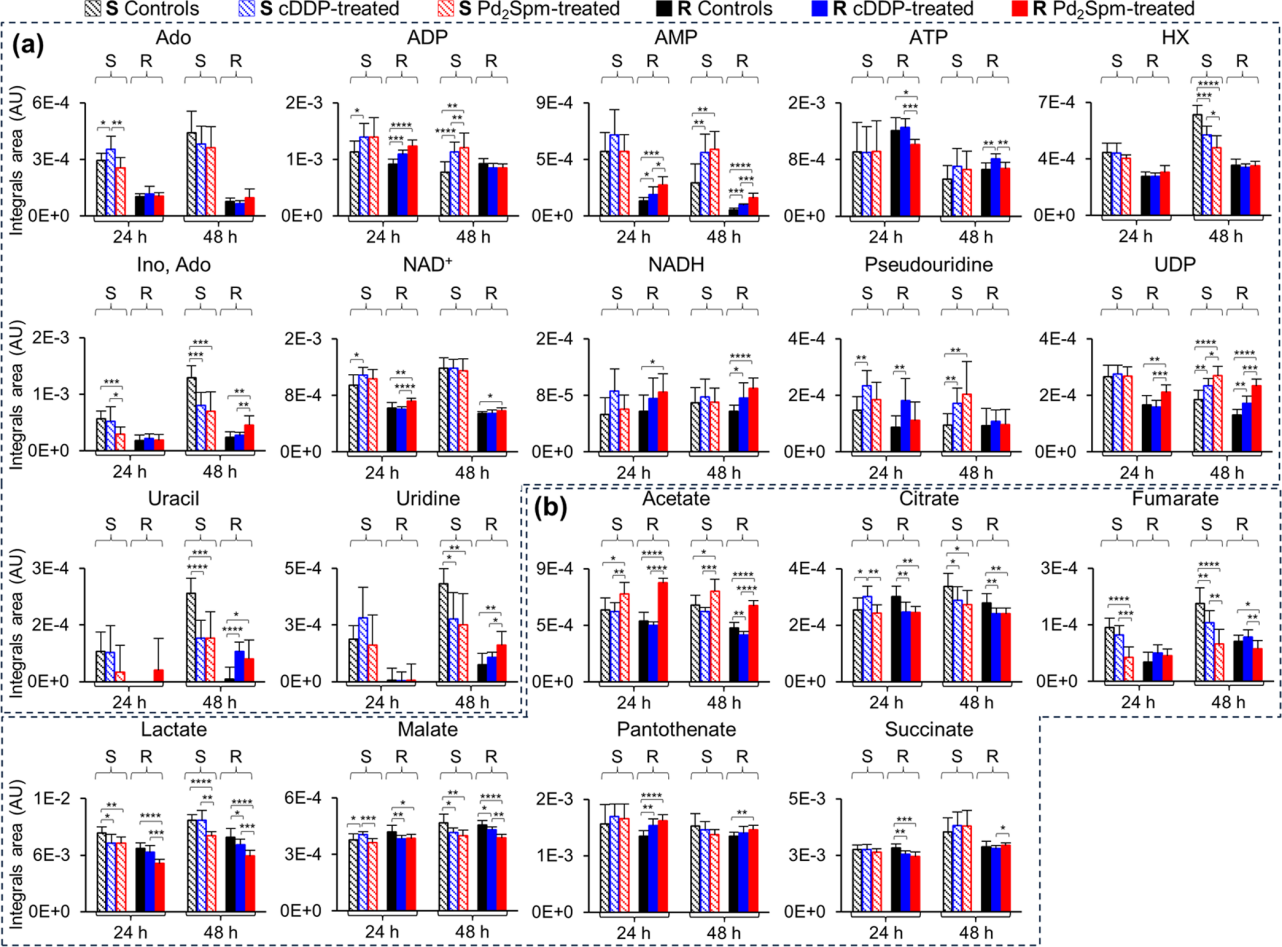
Time-course variations of selected (a) nucleotides and (b) organic acids in MDA-MB-231 (S, striped bars) and MDA-MB-231/R (R, full bars) cells, treated with PBS (controls, black), cDDP (blue), or Pd_2_Spm (red). Values are expressed as the mean of normalized area of integrated peak ± SEM. Abbreviations are defined in the captions of Figures 1–3. Significant differences between treated groups (R and S cells) and controls, at each time-point: *p-value <0.05; **p-value <0.01; ***p-value <0.001; ****p-value <0.0001.

The behavior of each amino acid is more clearly described in comparative plots for all groups and conditions (Figures 4a and S2 for metabolites varying more or less markedly, respectively). In these plots, the significant changes between treated and untreated samples are indicated (asterisks), whereas significant differences found in the direct comparison of S cells are listed in Table S2. Interestingly, R cells exhibit a higher glutamine/glutamate ratio than S cells under all conditions (Figure S3a), reflecting the inherently distinct glutamine and glutamate levels in R cells vs S cells (higher glutamine and lower glutamate in R cells; Figure 4a and Table S2). This ratio tends to decrease in cDDP-treated R cells (due to a decrease in glutamine) but not in S cells. Increased glycerophosphocholine (GPC) and phosphocholine (PC) appear to be a marker of R cells’ response to cDDP (compared to controls), as these metabolites do not change in S cells (Figure 3a). In R cells, this is reflected by generally higher PC/choline (Cho) ratios (due to the lower Cho levels in R cells; Figure 4 and Table S2). This ratio is further increased by the cDDP treatment (Figure S3c) due to the concomitant PC increase and Cho decrease (clearer in R cells than in S cells) (Figure 4b and Table S2). For nucleotides, the pattern of variations in R cells, upon cDDP exposure, is dominated by increases, with the exception of ATP (Figure 3a and Table S1). Notably, lower nucleotide levels generally characterize both untreated^28^ and cDDP-treated R cells, compared to S cells (Figures 5a and S2 and Table S2), with the exception of ATP and NADH levels (comparable between R and S). These features lead to lower ADP/ATP, AMP/ATP, and NAD^+^/NADH ratios in R cells, independently of treatment (Figure S3d–f). The effect of cDDP treatment is clearer at 48 h when R cells exhibit decreased ADP/ATP and NAD^+^/NADH ratios and slightly increased AMP/ATP (Figure S3d–f), while S cells only show a NAD^+^/NADH decrease (Figure S3f).

Other nucleotide differences between each cDDP-treated cell line and controls (Figure 3a) include (i) decreases in hypoxanthine (HX) and inosine and/or adenosine in S cells (unchanged in R cells), (ii) marked uracil decrease and increase in S and R cells, respectively, and (iii) increased uridine diphosphate-*N*-acetylglucosamine (UDP-GlcNAc) levels in R cells (unchanged in S cells).

The two cell lines share a time-dependent depletion in the tricarboxylic acid (TCA) cycle intermediates citrate and malate, with R cells showing no change in fumarate and an additional decrease in succinate (Figure 3a and Table S1). Notably, fumarate levels are kept lower in R cells than in S cells for both controls and cDDP-treated cells (Figure 5b), whereas the remaining detectable TCA intermediates (citrate, malate, and succinate) exhibit comparable levels for both cell lines. Both cDDP-treated cell lines suffer depletion in lactate and acetate at some point during treatment (Figure 3a and Table S1), with the levels of both acids being generally kept lower for R cells, compared to S cells (Figure 5b).

In addition, R cells respond to cDDP with an increase in pantothenate (PA), whereas this compound does not change significantly in S cells (Figure 3a and Table S1). CDDP-treated R cells show a marked decrease in dimethylamine (DMA) at 24 and 48 h (absent in S cells), no increase in glycerol (noted in S cells at 48 h), and an increase in *m-*inositol at 48 h (absent in S cells) (Figures 3a and S2 and Table S1).

### Metabolic Profiling of Pd_2_Spm Impact on cDDP-Resistant (R) and -Sensitive (S) Cells

**Figure 6:**
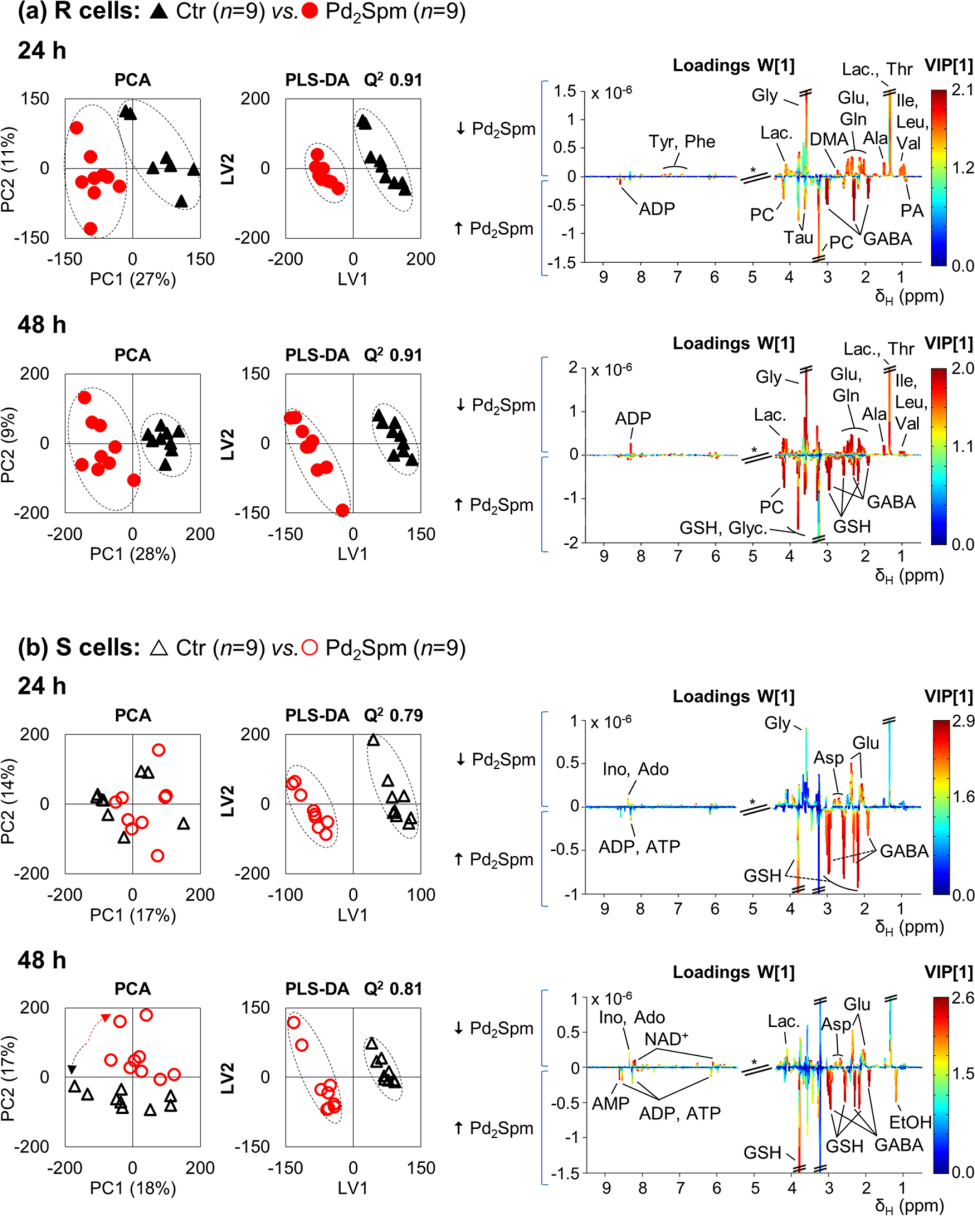
Pairwise PCA and PLS-DA scores and PLS-DA loading plots for ^1^H-NMR spectra of aqueous extracts of Pd_2_Spm-treated cells: (a) MDA-MB-231/R (R) and (b) MDA-MB-231 (S) cell lines. Pd_2_Spm-treated samples (red circles, *n* = 9) vs controls (black triangles, *n* = 9) at 24 and 48 h after treatment. Validation parameters (*R*^2^ and *Q*^2^) are shown for the PLS-DA model. Loading peak assignments are indicated for the most relevant metabolites, according to VIP. Abbreviations: EtOH, ethanol; other abbreviations as defined in the captions of Figures 1 and 2.

Multivariate analysis shows that the Pd(II) complex induces stronger metabolic responses in both R and S cells compared to cDDP, with visible PCA group separation for R cells (48 h) (Figure 6a) and S cells (24 and 48 h) (Figure 6b). PLS-DA models almost reach maximum predictive power (*Q*^2^ 1.0) for both cell lines, with a slightly higher impact of the complex on R cells (*Q*^2^ 0.91 for 24 and 48 h) compared to S cells (*Q*^2^ 0.79 and 0.81 for 24 and 48 h) (i.e., suggestive of a slightly stronger metabolic response of R cells). Compared to controls, effect size variations are indeed stronger for both Pd_2_Spm-treated R and S cells (Figure 3b and Table S3), with a tendency for generally more significant changes in R cells: amino acids (dominated by decreases but including strong GSH and GABA increases in both cell lines) (Figure 4a), choline compounds (again only varying (increasing) in R cells) (Figure 4b), nucleotides (with more pronounced increases in R cells) (Figure 5a), organic acids (with more pronounced decreases in TCA cycle intermediates and increases in acetate and PA, in R cells) (Figure 5b), and decreased DMA and *m*-inositol in R cells only (Figure 5).

**Figure 7:**
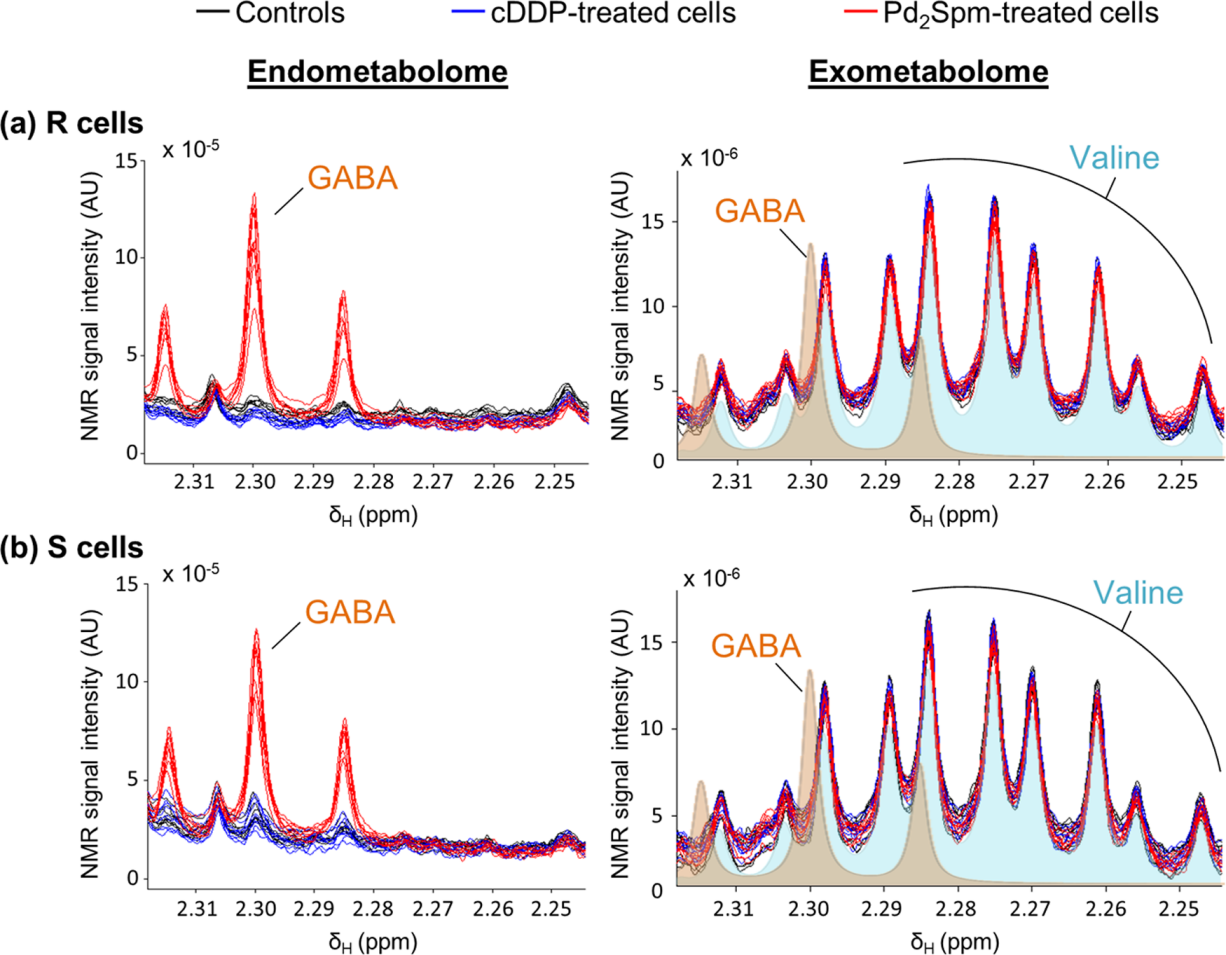
^1^H-NMR spectral regions (δ 2.24–2.32) of polar cell extracts (endometabolome, left) and cell media (exometabolome, right), illustrating intracellular GABA and its absence in extracellular media. (a) MDA-MB-231/R (R) and (b) MDA-MB-231 (S) cells treated with PBS (controls, black trace), cDDP (blue trace), or Pd_2_Spm (red trace).

In relation to amino acids, increased GABA and GSH levels appear to be specific to Pd_2_Spm action irrespective of cell line (Figures 3b and 4a and Table S3). Other amino acids are more strongly depleted in R cells than in S cells with no clear evidence of recuperation to control levels within 48 h (Figure 3b). Methionine remains unchanged from controls in Pd_2_Spm-treated R cells (while it was strongly decreased by cDDP in the same cells). Furthermore, Pd_2_Spm action decreases the glutamine/glutamate ratio in R cells (similarly to cDDP) (Figure S3), while specifically decreasing glutamate/GABA ratio (in R and S cells) as a reflection of the important GABA increase. To test if GABA exited the cells, we examined the NMR spectra of cell media (Figure 7a,b). The GABA triplet centered at δ 2.30, which is clearly prominent in the endometabolome of Pd_2_Spm-treated cells (Figure 7, left), is absent in the corresponding exometabolomes (Figure 7, right), as shown by the noncoincident superposition with the GABA profile (in orange) and the perfect coincidence with the blue outline indicating the presence of valine. Notably, although the complete absence of GABA could not be confirmed by 2D NMR due to low signal intensity, the symmetry of the valine profile rules out any significant overlap with underlying GABA peaks.

Other Pd_2_Spm-induced changes include increased choline (in R cells only) (Figures 3b and 4), although treatment does not significantly change the PC/Cho ratio in either cell line, in contrast to cDDP, which was seen to increase the PC/Cho ratio (Figure S3). In addition, Pd_2_Spm specifically induces (i) increases in adenine (R cells), IMP (S cells), inosine/adenosine (R cells), uridine monophosphate (UMP) (R and S cells), uridine (R cells), and acetate (R and S cells) and (ii) decreases in ATP and *m*-inositol (R cells) (Figures 5a and S2). In particular, nucleotide variations lead to increased ADP/ATP and AMP/ATP ratios and a decreasing trend in NAD^+^/NADH (Figure S3), which is more clearly seen in Pd_2_Spm-treated R cells.

## Discussion

### cDDP Impact on MDA-MB-231 (S) and MDA-MB-231/R (R) Cell Metabolome

The cytotoxic impact of cDDP on S cells is expressed by an IC_50_ of 1.0 μM (48 h),^20^ conditions under which a significant extent of cell death has been detected. Our results showed that the corresponding metabolite signature of cDDP-treated S cells includes a relatively minor disturbance of amino acid metabolism, although more enhanced at 48 h than at 24 h. At 48 h, sensitive cells are depleted in aspartate and lysine (which may be entering the TCA through oxaloacetate and acetyl-CoA, respectively) and NAA. NAA (expected to arise from *N*-acetyl-aspartyl-glutamic acid (NAAGA)) has been suggested to promote oxidative stress and lipid/protein oxidation.^29^ We propose that lower NAA levels upon cDDP treatment may evidence less oxidative stress due to activated antioxidant protection mechanisms. This is consistent with an additional decrease in GSH and an increase in taurine (oxidized form of hypotaurine), suggesting the involvement of both GSH/GSSG and hypotaurine/taurine pairs, with a concomitant decrease in HX (probably through oxidation to xanthine). This protection appears to reduce the redox status of cells upon cDDP exposure, as expressed by the NAD^+^/NADH decrease. This ratio is generally higher for S than for R cells, suggesting that, despite activated protective mechanisms, the former still experience higher oxidative stress, as previously proposed.^30^ High glutaminolytic activity is a known feature of cancer cells,^31,32^ and the globally lower glutamine/glutamate ratio (more active glutaminolysis) in S than in R cells was not affected by cDD. This suggests that the Pt(II) drug does not affect this pathway in S cells. The Pt(II) drug also does not seem to change the ATP energy status (no change in ADP/ATP and AMP/ATP), although creatine (Cr)/PCr energy metabolism is slightly disturbed. However, both ADP/ATP and AMP/ATP are kept higher (higher expenditure of ATP) in S cells, both untreated and cDDP-exposed, in relation to R cells. We hypothesize that ATP may be used more extensively in S cells to keep the membrane metabolism stable. This is consistent with the absence of changes in membrane PL precursors (namely, Cho, PC, or GPC) upon cDDP treatment of S cells.

We also postulate that ATP in S cells may be involved in pyrimidine metabolism, especially increasing pseudouridine and uridine diphosphate (UDP), at the expense of uracil and uridine. These may be related to RNA modification and enhanced glycosylation processes, respectively.^33,34^ Exposure to cDDP slightly lowers lactate levels (at 24 h, but not at 48 h). This may reflect a preliminary shift toward less glycolytic activity and preferential use of pyruvate to feed the TCA cycle. This would support the interpretation of depletion in TCA intermediates as evidence of increased cycle activity, also supporting the anaplerotic use of aspartate and lysine mentioned above. Increased glycerol in cDDP-treated S cells may indicate some lipid oxidation, consistent with higher oxidative stress. Hence, we propose that S cells under the action of cDDP experience high oxidative stress and mobilize GSH, taurine, and HX (and, possibly, NAA) for oxidative protection and subsequent reduction of NAD^+^/NADH. CDDP-treated S cells maintain the inherently high glutaminolytic activity, as well as high ADP/ATP and AMP/ATP ratios (reflecting high ATP requirements) and an effective membrane metabolism balance, as in untreated cells.^28^ The high ATP consumption in the presence of cDDP (ATP probably arising through the increased TCA cycle activity and lipid oxidation) may be related to meeting the energy demands of membrane PL biosynthesis and pyrimidine metabolism.

CDDP treatment of R cells is characterized by more than one order of magnitude higher IC_50_ (32.4 μM),^27^ which clearly reflects resistance of the cells to cDDP treatment. The metabolic profile of R cells is more responsive to cDDP than that of S cells, which may reflect some kind of protective behavior against the drug to avoid/reduce cell death. The early (24 h) strong demand of many amino acids, largely recovered at 48 h, may indicate an increased need for protective (antioxidative) enzymes and/or anaplerotic feeding of the TCA cycle for increased ATP production. ATP is indeed seen to increase at 48 h, as expressed by a lower ADP/ATP ratio. Glutaminolytic activity (inherently lower in R cells compared to that in S cells) is enhanced by cDDP on R cells, whereas lactate depletion also occurs (as in S cells), both observations being in agreement with enhanced TCA cycle activity. In turn, this is supported by slightly stronger depletions in citrate, malate, and succinate. Interestingly, the absence of changes in GSH, taurine, and HX suggests that no/low oxidative stress protection characterizes cDDP-treated R cells, inherently characterized by low oxidative stress. However, cDDP does induce a small NAD^+^/NADH reduction (48 h), which indicates that some extent of oxidative protection is occurring but not as importantly as in S cells. Adenine is reduced in cDDP-treated R cells, probably reflecting a distinct ADP/AMP/ATP balance. Also, pyrimidine metabolism is differentially affected, suggesting a distinct glycosylation status, consistent with the increase in UDP-GlcNAc in R cells and not in S cells. Furthermore, an initial pseudouridine increase is recovered at 48 h, suggesting that R cells may have a greater capacity for RNA repair than S cells. Increased PA (vitamin B5), a precursor of CoA and of the acyl-carried protein (ACP),^35^ could serve as an additional source of CoA to enhance TCA and ATP production.

However, the decrease in acetate and absence of glycerol changes (contrary to that observed in S cells) lead to the hypothesis that both PA (through ACP) and acetate may be supporting enhanced lipid biosynthesis in R cells, rather than oxidation. This is a possible sign of resistance to membrane degradation as a response to cDDP. We suggest that the enhanced anaplerotic activity of amino acids may render lipid oxidation unnecessary to attain the required higher ATP levels (and lower ADP/ATP ratio). PL metabolism responds differently in R cells, with increased PC and GPC levels (unchanged in S cells). Although this could be seen as evidence of membrane degradation, the fact that PC/Cho is consistently higher in cDDP-treated R cells may also be interpreted as a higher capacity for PL biosynthesis. This is in agreement with the supportive roles of PA and acetate suggested above, perhaps to promote cell proliferation or cell membrane repair. However, the precise origin of the choline compound profile that characterizes cDDP-treated R cells requires further investigation. Hence, cDDP treatment of R cells results in a metabolic “resistance signature” that includes significantly increased TCA cycle activity supported by amino acids (including enhanced glutaminolysis) and decreased lactate fermentation (the latter in common with cDDP-treated S cells) to increase ATP levels. In contrast with S cells, treated R cells appear to increase the level of lipid biosynthesis (possibly for PL synthesis to support cell proliferation) and exhibit a distinct pyrimidine metabolic pattern (possibly related to improved RNA repair mechanisms). Interestingly, there is no evidence of high oxidative stress (or significant protective mechanisms) in R cells (as there was in S cells), although cDDP does have a slightly lower redox status (even if this is already much lower in R cells than in S cells).

### Pd_2_Spm Impact on MDA-MB-231 (S) and MDA-MB-231/R (R) Cell Metabolome

The cytotoxicity of Pd_2_Spm on S cells (IC_50_ 7.9 μM) is in broad agreement with that of cDDP (1.0 μM) on the same cells,^21^ supporting the potential of the Pd(II) complex as a promising new drug (at least against cDDP-sensitive cells). However, a more rapid and significant response of amino acids to the Pd(II) complex (depletion of 13 anaplerotic amino acids, with no recovery at 48 h) and a stronger fumarate depletion suggest a more immediate trigger of TCA activity than in cDDP/S cells (where amino acid depletion is only seen at 48 h). Interestingly, this does not change glutamine/glutamate ratios (as also seen in cDDP/S cells). Apart from the maintenance of glutaminolytic activity, other similarities between cDDP/S cells and Pd_2_Spm/S cells include no detectable changes in membrane metabolism or in ADP/ATP and AMP/ATP ratios (along with similar changes in Cr metabolism) and a number of similar nucleotide features (namely, decreased inosine/adenosine, uracil, and uridine and increased ADP, AMP, pseudouridine, UDP, and UMP). We hypothesize that the above similarities may be associated with the high cytotoxicity effects of both cDDP and Pd_2_Spm on S cells (IC_50_ of 1.0 and 7.9 μM, respectively),^21^ particularly regarding the above nucleotide signature. This may reflect a similar effect of cDDP/Pd_2_Spm on nucleic acid damage–repair mechanisms and glycosylation processes.

With regard to the differences between Pd_2_Spm and cDDP treatment on S cells, these include (i) reduced lactate production (decreased Warburg effect, as with cDDP but more effective with the Pd(II) complex, which may be considered a beneficial aspect of the latter) and (ii) slightly increased acetate (but not glycerol), suggesting some degree of lipid oxidation, although not reflected in significant changes in membrane metabolism. Redox status (NAD^+^/NADH) of Pd_2_Spm-treated S cells remains high and equivalent to that of untreated cells. This is contrary to cDDP, which decreased redox status slightly. Indeed, in Pd_2_Spm/S cells, a different interplay of GSH/NAA seems to be involved (apparently with no involvement of taurine), and this will be further discussed below. Additionally, the absence of variation in ADP/ATP and AMP/ATP ratios found in Pd_2_Spm/S cells (remaining elevated in S cells compared to that in R cells) suggests that any lipid-oxidation-derived ATP may be promptly consumed to maintain membrane balance and pyrimidine and inosine metabolisms. This is expressed by more enhanced levels of UMP and UDP and an increase in IMP seen only in Pd_2_Spm/S cells. The increase in UMP may be related to the overexpression of uridine–cytidine kinase (CMPK1), a rate-limiting enzyme involved in the pyrimidine salvage pathway and previously associated with poor prognosis in TNBC patients.^36^ However, UMP may also be in demand for phosphorylation of UDP for subsequent involvement in glycosylation processes. Thus, the exact function of enhanced UMP levels requires further investigation. The increase of IMP in Pd_2_Spm/S cells, probably at the expense of inosine, may promote the activity of TCA enzymes as recently suggested,^37^ contributing to the enhanced anaplerosis discussed previously. GABA and GSH increases in Pd_2_Spm-treated R and S cells are clear markers of Pd_2_Spm, and as this appears to be independent of cell-sensitive/resistance characteristics, it will be discussed below for both cell lines together.

The cytotoxicity of Pd_2_Spm on R cells (IC_50_ 10.6 μM) is promisingly higher than that of cDDP in the same cells (32.4 μM).^27^ It is noteworthy that the metabolic impact of Pd_2_Spm on R cells is stronger compared to that of S cells, for instance, in terms of amino acid depletion at 24 h, with hardly any recovery of amino acid levels at 48 h (in contrast with cDDP/R cells). This may indicate that amino acid metabolism in R cells is not as resistant to Pd_2_Spm as it seems to be in relation to cDDP at 48 h. In addition, as the amino acid signature of Pd_2_Spm/R cells includes the changes noted in S cells as possibly indicating a positive response to treatment (namely, decreased aspartate, glutamate, lysine, NAA, and PCr and increased taurine), we postulate that such behavior in R cells treated with Pd_2_Spm may reflect the higher cytotoxicity of the Pd(II) complex against R cells. The decrease in glutamine/glutamate seen for cDDP/R cells is reproduced for Pd_2_Spm/R cells, with the effect thus seeming to be independent of the metal agent and a characteristic of R cells themselves. Besides the entry of amino acids into the TCA cycle, we suggest that the biosynthesis of protective proteins against enhanced oxidative stress may also occur in Pd_2_Spm/R cells. This is supported by increased GSH and taurine (perhaps related to increased *m*-inositol, also reported to have antioxidant activity),^38,39^ which may work to significantly reduce the NAD^+^/NADH ratio, in contrast with Pd_2_Spm/S cells (where such mechanism did not seem as efficient). Similarly to cDDP/R cells, increased levels of choline compounds are observed in Pd_2_Spm/R cells; such a signature seems to be a general characteristic of R cells, irrespective of the treatment. However, Cho, GPC, and PC are more markedly increased upon Pd_2_Spm, although no change in PC/Cho was observed (contrary to that seen in cDDP/R cells). Hence, the precise interplay of these precursors to balance PL biosynthesis and degradation of membrane lipids seems drug-dependent, and as PC/Cho is not increased by Pd_2_Spm, we suggest that PL biosynthesis may not be as enhanced in Pd_2_Spm/R cells as in Pd_2_Spm/S cells. This supports a predominant effect of membrane degradation in Pd_2_Spm/R cells, consistent with Pd_2_Spm higher cytotoxicity in R cells. Indeed, the more enhanced increases in acetate and PA in Pd_2_Spm/R cells are consistent with some extent of lipid oxidation, despite the lower redox status of R cells and contrary to cDDP/R cells for which we postulated a dominant role of lipid synthesis (probably of PLs).

Pd_2_Spm induces further important changes in R cells, namely, additional and/or more pronounced changes in adenine and uracil derivatives, all of which increase, except for ATP. The large increases in ADP/ATP and AMP/ATP ratios are a clear characteristic of Pd_2_Spm/R cells, where ATP seems to be extensively required for cell survival. Apart from the biosynthesis of antioxidant protective proteins suggested above, we hypothesize that ATP may also be supporting the altered status of nucleotide metabolism. Moreover, Pd_2_Spm induces a greater depletion in lactate in R cells, implying that the Pd(II) complex is more efficient in decreasing the Warburg effect in R cells than cDDP.

Finally, marked increases in GABA (undetected in untreated and in cDDP-treated cells) and GSH (increased by Pd_2_Spm and decreased by cDDP) are striking effects of Pd_2_Spm on both S and R cells. Increased GABA is an issue of recognized importance in determining the prognosis of breast cancer and in particular of TNBC. Indeed, increased GABA (either of exogeneous and/or endogenous origins) triggers a complex tumor-promoting GABAergic signaling network, increasing proliferation, and metastatic potential.^40−43^ It has been shown in MDA-MB-231 cells (and hence possibly in Pd_2_Spm/S cells) that intracellular GABA may originate from glutamate;^42^ however, glutamine/glutamate ratios are maintained in S cells. We hypothesize that a more likely origin for GABA is biogenic polyamine metabolism,^44,45^ here expected to be affected by the Pd_2_Spm complex itself. Indeed, the effects of both cDDP and Pd_2_Spm on the expression of genes related to polyamine metabolism (including the gene spermine, spermidine N 1-acetyltransferase (SSAT)) in ovarian cancer cells^18^ unveiled a distinct behavior of the palladium complex, in tandem with decreased intracellular biogenic amine levels. At this stage, the relationship of such effects with GABA levels remains unclear, although our results support the hypothesis that GABA may result from spermine carried in Pd_2_Spm. In fact, intracellular spermidine and putrescine have been reported to give rise to GABA via the enzymatic action of amiloride binding protein 1 (ABP), also known as diamine oxidase (involved in the intermediate step of 4-aminobutanal formation), and aldehyde dehydrogenase 9 A1 (involved in the conversion of 4-aminobutanal in GABA).^45^ The excess GABA synthesized in this pathway could then enter the TCA cycle via succinate through the action of GABA-transaminase (also known as 4-aminobutyrate aminotransferase, ABAT), although ABAT expression is known to depend on breast cancer subtype.^45,46^ However, if this is the case, it does not result in a relevant succinate increase in either cell type, thus making this intracellular GABA end point insufficient to explain the impact of its marked increase. Of course, a strongly undesirable outcome would arise if GABA leaves from the intracellular medium into the extracellular matrix, where it is known to induce associated tumor-promoting properties and increased metastatic potential.^51^ However, we have demonstrated that GABA is, surprisingly, retained in the intracellular environment of Pd_2_Spm-treated R/S cells. Since Na^+^/K^+^-ATPase has been identified as an important GABA transporter, mostly studied in brain but known to be a ubiquitous transmembrane protein,^47−49^ we postulate that its function in MDA-MB-231 cells may be somehow subdued in Pd_2_Spm-treated cells. This ATPase pumps K^+^ from the extracellular medium into the cells, in exchange for the extrusion of Na^+^, while hydrolyzing intracellular ATP.^47^ ATP availability is, therefore, a determinant for the adequate function of this controlled translocation of sodium and potassium ions across the cell membrane. We recall that in Pd_2_Spm/S cells, ADP/ATP and AMP/ATP ratios remain higher than in Pd_2_Spm/R cells, reflecting lower availability of ATP (due to its possible engagement in sustaining membrane balance, pyrimidine metabolism, and inosine metabolism). In addition, in Pd_2_Spm/R cells, large increases in ADP/ATP and AMP/ATP ratios upon treatment indicate higher ATP requirements relative to untreated cells (suggested above to involve the biosynthesis of antioxidant protective proteins and altered status of nucleotide metabolism). Thus, we hypothesize that the use of ATP toward other ends (in S cells generally and in R cells particularly under Pd_2_Spm treatment) might render ATP levels too low for the adequate function of Na^+^/K^+^-ATPase. The question remains as to the possible end points of the excess intracellular GABA in Pd_2_Spm-treated cells. It is possible that GABA transamination will occur through ABAT to yield succinil-semialdehyde (SSA), along with alanine or glutamate (depending on the nature of the active substrate: pyruvate or α-ketoglutarate, respectively), all of these metabolites being able to enter the TCA cycle.^45^ However, there is interesting recent evidence that increased GABA levels are associated with antioxidant mechanisms in a wide range of organisms.^50,51^ This is indeed consistent with the concomitant marked increases in GSH, along with GABA, in all Pd_2_Spm-treated cells as well as with the more marked depletion in NAA (lower NAA levels suggested earlier to evidence less oxidative stress due to activated oxidative stress protection mechanisms). We suggest that simultaneously high GSH and low NAA levels may indicate a more efficient protective mechanism in Pd_2_Spm-exposed cells (with or without the involvement of taurine or HX, as these are not always seen to change), probably related to the high and comparable cytotoxicity of Pd_2_Spm in both S (IC_50_ = 7.9 μM) and R cells (IC_50_ = 10.6 μM). We therefore advance the hypothesis that the Pd_2_Spm-mediated increase in intracellular GABA may actually translate into a beneficial antioxidant effect, the exact mechanism of which necessarily requires further important investigation.

## Conclusions

This work investigated the metabolic response of cDDP-sensitive (S) and -resistant (R) MDA-MB-231 cell lines to exposure to cDDP itself (establishing signatures of response and resistance) and to Pd_2_Spm (unveiling metabolic deviations probably associated with its high cytotoxicity against both cell lines). Exposure of S cells to cDDP led to enhanced antioxidant protective mechanisms, able to decrease the inherently high redox status (NAD^+^/NADH) of S cells, compared to R cells (while inherent higher glutaminolytic activity and ADP/ATP and AMP/ATP ratios were not affected). Slight deviations were induced in the TCA cycle activity (enhanced), pyrimidine metabolism, and lipid oxidation (enhanced) while leaving membrane metabolic profile unaffected. Conversely, R cell metabolism responded strongly to cDDP, supporting a proposed “resistance signature”, which comprised further activated TCA cycle (amino acid/lactate depletion and activated glutaminolysis), adapted AMP/ADP/ATP status, enhanced PL biosynthesis (probably for cell proliferation and/or cell membrane repair), deviant adenine/uracil fingerprints, and, interestingly, no evidence of effective oxidative stress protection. Hence, cDDP *in vitro* resistance of MDA-MB-231 cells seems to involve larger metabolic plasticity impacting on TCA cycle activity, AMP/ADP/ATP pools, lipid and membrane metabolisms, and adenine/uracil metabolism (without significant oxidative stress effects).

Pd_2_Spm impacted the metabolism of both R and S MDA-MB-231 cells more markedly than cDDP. Similarities between Pd_2_Spm/S cells and cDDP/S cells (no changes in glutaminolytic activity, membrane metabolism, and AMP/ADP/ATP pools, and similar adenine/uracil derivatives patterns) are putatively associated with high cDDP/Pd_2_Spm cytotoxicity upon S cells. However, marked differences arose in Pd_2_Spm/S cells, including a more immediate TCA activity trigger, distinct uracil/inosine metabolisms, and prominent increases in GABA and GSH (common to Pd_2_Spm/R cells). The impact of Pd_2_Spm on R cells was more extensive (enhanced TCA activity, changed AMP/ADP/ATP pools, apparent membrane degradation, and altered nucleotide profile), also including features observed in cDDP/S cells as possible indicators of high cytotoxicity.

The above results suggest Pd_2_Spm as effectively triggering metabolic effects possibly associated with higher cytotoxicity in S cells and, more importantly, in resistant MDA-MB-231 cells. However, Pd_2_Spm also gave rise to high levels of intracellular GABA, generally known to play several undesirable roles in the cancer microenvironment once it exits cancer cells. In treated R/S cells, however, GABA did not exit the cells (perhaps due to ATP unavailability for the adequate function of Na^+^/K^+^-ATPase) and we hypothesize that the excess intracellular GABA may exert a beneficial antioxidant function, also involving GSH and NAA. Overall, these results illustrate the usefulness of untargeted NMR metabolomics in contributing to the understanding of cDDP sensitivity and resistance in MDA-MB-231 cells and of why Pd_2_Spm may be a possible alternative to treat cDDP-resistant TNBC.

## Experimental Section

### Chemicals and Solutions

CDDP (*cis*-dichlorodiammine platinum(II), 99.9%), potassium tetrachloropalladate (II) (K_2_PdCl_4_, 98%), spermine (*N,N’*-bis(3-aminopropyl)-1,4-diaminobutane, 99%), Dulbecco’s modified Eagle’s medium—high-glucose cell growth medium (DMEM-HG), trypan blue (0.4% w/v), and trypsin-EDTA (1×), as well as inorganic salts and acids were all purchased from Sigma-Aldrich Chemical S.A. (Sintra, Portugal). Fetal bovine serum (FBS) was obtained from Gibco-Life Technologies (Porto, Portugal). The Pd_2_Spm complex was synthesized according to published procedures.^52,53^ The newly synthesized compound was characterized (and tested for purity) by ^1^H NMR and Fourier transform infrared (FTIR) spectroscopy. The drugs’ solutions were freshly prepared by dissolving an appropriate quantity of drug in phosphate-buffered saline, PBS (H_2_PO_4_ 1.5 mM, Na_2_HPO_4_ 4.3 mM, KCl 2.7 mM, NaCl 150 mM, pH 7.4). Stock solutions of cDDP (10 μM) and Pd_2_Spm (79 μM) were prepared, sterile-filtered (ø 0.22 μm), and stored at −20 °C. All reagents/products were of analytical grade, and when available for purchase, they were >95% pure by HPLC analysis.

### Cell Culture

The human TNBC cell line MDA-MB-231 (ATCC HTB-26; absence of estrogen and progesterone receptors, HER2 overexpression) was purchased from ATCC (Manassas, VA). MDA-MB-231 cells were cultured in DMEM-HG cell growth medium supplemented with 10% (v/v) FBS and maintained under a humidified atmosphere of 5% CO_2_ at 37 °C. The cDDP-resistant cell line was established as previously described (exposure of MDA-MB-231 cells to increasing concentrations of cDDP, up to a maximum of 2 μM, during 6 months), and it was designated as MDA-MB-231/R.^27^ Cell population doubling times were 25.5 ± 0.9 and 30.6 ± 1.1 h for MDA-MB-231 and MDA-MB-231/R cells, respectively. Cell cultures were routinely screened for mycoplasma contamination, with all assays having yielded negative results.

### Cell Exposure and Collection

MDA-MB-231 parental (cDDP-sensitive, S) and resistant (R) cells were seeded at a density of 3 × 10^4^ cells/cm^2^ onto 150 mm Petri dishes, cultured in a humidified atmosphere of 5% CO_2_ at 37 °C, and allowed to adhere for 24 h. After this time, the experiment was initiated by adding stock solutions of each drug to achieve the corresponding half of maximal inhibitory effect IC_50_ values, previously determined for S cells: 1.0 μM cDDP and 7.9 μM Pd_2_Spm.^21^ The cells were then incubated and collected at 24 and 48 h, with a basis on the population doubling times mentioned above. At each time-point, cells were harvested using a 0.25% (v/v) trypsin-EDTA solution (1×), washed with PBS, and centrifuged (300*g*, 5 min, 20 °C) twice. The cell pellet was immediately stored at −80 °C until analysis. In addition, cell media were also collected at each time-point subsequently following a protein-precipitation treatment, before NMR analysis of selected metabolites.^54^ Three independent experiments with triplicate were performed for each cell line, giving a total of *n* = 9 for each condition (Figure S4).

### Cell Extraction

Cellular polar extracts were obtained using a methanol/chloroform/water biphasic extraction method.^55^ Briefly, cell pellets were resuspended in 650 μL of 80% (v/v) methanol-MilliQ water solution, transferred to microcentrifuge tubes with 150 mg of glass beads (ø = 0.5 mm), and vortexed for 5 min to aid cell disruption. Subsequently, (i) 260 μL of 100% chloroform and (ii) 260 μL of 100% chloroform plus 220 μL MilliQ water were added to samples, which were then vortexed for 5 min between solvent addition. Samples were stored at −20 °C for 10 min and centrifuged (2000*g*, 15 min, room temperature). The aqueous phase was collected into a new tube, vacuum-dried, and stored at −80 °C until further analysis. All samples and reagents were kept on ice during the extraction procedure. Before NMR analysis, the dry aqueous extracts were suspended in 650 μL of 100 mM sodium phosphate buffer (pH 7.4, in D_2_O containing 0.25% 3-(trimethylsilyl)-propionic-2,2,3,3-d4 acid (TSP) for chemical shift referencing) and transferred into 5 mm NMR tubes.

### NMR Spectroscopy and Statistical Analysis of Spectra

The NMR spectra were recorded at 298 K on a Bruker AVANCE III spectrometer equipped with a 5 mm TXI probe, operating at 500.13 MHz for ^1^H observation. Standard 1D ^1^H-NMR spectra of aqueous extracts (and selected media samples) were acquired using a water presaturation pulse sequence (“noesypr1d” from Bruker library, Rheinstetten, Germany), with a 7002.801 Hz spectral width, 32 k data points, a 2.34 s acquisition time, and a 2 s relaxation delay and 512 scans. Prior to Fourier transformation, each free-induction decay was zero-filled to 64 k points and multiplied by a 0.3 Hz exponential line-broadening function. Spectra were manually preprocessed including phase correction, baseline adjustment, and internal calibration of chemical shifts to TSP. For peak assignment, 2D NMR homonuclear total correlation (TOCSY) and heteronuclear single-quantum correlation (HSQC) spectra were acquired for selected samples, along with comparison with the existing literature and spectral databases, such as Bruker BIOREFCODE (AMIX-viewer 3.9.14, Bruker Biospin, Rheinstetten, Germany), human metabolome database (HMDB),^56^ and Chenomx NMR Suite (Chenomx Inc., Edmonton, AB, Canada).^56^ Additionally, the assignment of selected peaks was confirmed by statistical total correlation spectroscopy (STOCSY) (Matlab 8.3.0, The MathWorks Inc., Natick, MA).^57^ The 1D ^1^H NMR spectra were converted into matrices (AMIX 3.9.14, Bruker Biospin, Rheinstetten, Germany), excluding methanol (δ 3.36, singlet) and water (δ 4.4–5.4) spectral regions. The spectra were aligned by recursive segment-wise peak alignment (RSPA) to minimize chemical shift variations (Matlab 8.3.0, The MathWorks Inc., Natick, Massachusetts) and normalized to the total spectral area to account for different cell numbers. Multivariate analysis was carried out using principal component analysis (PCA) and partial least-squares-discriminant analysis (PLS-DA), after unit variance (UV) scaling (SIMCA-P 11.5; Umetrics, Umeå, Sweden). PLS-DA models with values of predictive power (*Q*^2^) higher than 0.50 were considered eligible for further analysis. PLS-DA loadings were back-transformed, multiplying each variable by its standard deviation and colored according to variable importance to the projection (VIP) (Matlab 8.3.0, The MathWorks Inc., Natick, MA). Loading plots revealed candidate resonances relevant for class separation, which were selected for area integration (AMIX 3.9.14, Bruker BioSpin, Rheinstetten, Germany), normalization, and variation assessment by univariate analysis. Univariate analysis of metabolites included effect size (ES) and statistical significance calculation (Shapiro–Wilk test to assess data normality, Student’s *t* test, or Wilcoxon test for normally distributed or non-normally distributed data, respectively) (R statistical software).^58^ For multiple testing, *p*-values of significantly changed metabolite levels (|ES| > ES error and *p* < 0.05) were corrected by false discovery rate (FDR), based on the Benjamini and Hochberg method.^59^ Significant metabolite differences were confirmed by visual inspection of the spectra and putatively interpreted based on the literature and the Kyoto Encyclopedia of Genes and Genomes (KEGG) database.^60^

## Supporting Information Available

Figure S1. Average 500 MHz ^1^H-NMR spectra of aqueous extracts of cDDP-sensitive MDA-MB-231 cells (a) untreated and treated with (b) cDDP and (c) Pd_2_Spm after 48 h; Figure S2. Bar charts illustrating time-course variations of (a) amino acids, (b) nucleotides, and (c) other compounds significantly changed in MDA-MB-231 and MDA-MB-231/R cells; Figure S3. Bar charts illustrating time-course variations of ratios (a) Gln/Glu, (b) Glu/GABA, (c) PC/Cho, (d) ADP/ATP, (e) AMP/ATP, and (f) NAD^+^/NADH, calculated for MDA-MB-231 and MDA-MB-231/R cells; Figure S4. Workflow illustrating the treatment protocol of triple-negative breast cancer cDDP-sensitive (MDA-MB-231) and -resistant (MDA-MB-231/R) cell lines; Table S1. Statistically significant metabolite variations observed in MDA-MB-231 cDDP-sensitive (S) and -resistant (R) cells treated with cDDP, compared to controls; Table S2. Statistically significant metabolite variations observed in MDA-MB-231/R (R) cells directly compared to MDA-MB-231 (S) cells, considering controls (left), cDDP-treated (middle), and Pd_2_Spm-treated (right) groups at 48 h only; and Table S3. Statistically significant metabolite variations observed in MDA-MB-231 cDDP-sensitive (S) and -resistant (R) cells treated with Pd_2_Spm, compared to controls (PDF)

## Abbreviations

Ac.: acetate
ACP: acyl-carried protein
Ado: adenosine
ADP: adenosine diphosphate
AMP: adenosine monophosphate
ATP: adenosine triphosphate
BC: breast cancer
BCAAs: branched-chain amino acids
cDDP: cisplatin
CDX: cell-derived xenograft
Cho: choline
Cr: creatine
DMA: dimethylamine
ES: effect size
FBS: fetal bovine serum
FDR: false discovery rate
Fum.: fumarate
GABA: γ-aminobutyrate
Glyc.: glycerol
GPC: glycerophosphocholine
GSH/GSSG: glutathione (reduced/oxidized forms)
HX: hypoxanthine
IMP: inosine monophosphate
Ino: inosine
*m*-Ino: *myo*-inositol
Lac.: lactate
NAA: *N*-acetyl-aspartate
NAD^+^/NADH: nicotinamide adenine dinucleotide (oxidized/reduced)
NMR: nuclear magnetic resonance
PA: pantothenate
PBS: phosphate-buffered saline
PC: phosphocholine
PCA: principal component analysis
PCr: phosphocreatine
Pd_2_Spm: palladium-spermine complex
PLs: phospholipids
PLS-DA: partial least-squares-discriminant analysis
Pseudourd.: pseudouridine
*Q*^2^: predictive power
Spd: spermidine
Spm: spermine
Tau: taurine
TCA: tricarboxylic acid cycle
TNBC: triple-negative breast cancer
UDP: uridine diphosphate
UDP-Glc/GlcA: uridine diphosphate-glucose/glucuronate
UDP-GlcNAc: uridine diphosphate-*N*-acetylglucosamine
UMP: uridine monophosphate
VIP: variable importance to the projection
FTIR: Fourier transform infrared
HMDB: human metabolome database
INS: inelastic neutron scattering
KEGG: Kyoto Encyclopedia of Genes and Genome
TSP: 3-(trimethylsilyl)-propionic-2,2,3,3-d4 acid
UV: unit variance
