# Supplementary material for: Pd2Spermine as an Alternative Therapeutics for Cisplatin-Resistant Triple-Negative Breast Cancer
Source: J Med Chem. 2024 Apr 9;67(8):6839–53. doi: 10.1021/acs.jmedchem.4c00435 (PMC11056979; doi:10.1021/acs.jmedchem.4c00435)
Supplement: Supplementary file 1 — jm4c00435_si_001.pdf [file jm4c00435_si_001.pdf]

SUPPORTING INFORMATION

Pd_2Spermine as an alternative therapeutics for cisplatin-resistant triple-negative breast cancer

Tatiana J. Carneiro [†,‖,‡] , Ana L. M. Batista de Carvalho [‖], Martin Vojtek [‡], Raquel C. Laginha [‖], Maria Paula M. Marques [‖,§], Carmen Diniz [‡,*] and Ana M. Gil [†,*]

[†] Department of Chemistry and CICECO – Aveiro Institute of Materials, University of Aveiro, 3810-193 Aveiro, Portugal

[‖] Molecular Physical-Chemistry R&D Unit, Department of Chemistry, University of Coimbra, 3004-535 Coimbra, Portugal

[‡] LAQV/REQUIMTE, Laboratory of Pharmacology, Department of Drug Sciences, Faculty of Pharmacy, University of Porto, 4150-755 Porto, Portugal

[§] Department of Life Sciences, Faculty of Science and Technology, University of Coimbra, 3000-456 Coimbra, Portugal

Figure S1. Average 500 MHz ^{1}H-NMR spectra of aqueous extracts of cDDP-sensitive MDA-MB-231 cells (a) untreated and treated with (b) cDDP and (c) Pd_2Spm, after 48 h.

Figure S2. Bar charts illustrating time-course variations of **(a)** amino acids, **(b)** nucleotides and **(c)** other compounds significantly changed in MDA-MB-231 and MDA-MB-231/R cells.

Figure S3. Bar charts illustrating time-course variations of ratios (a) Gln/Glu, (b) Glu/GABA, (c) PC/Cho, (d) ADP/ATP, (e) AMP/ATP and (f) NAD^+/NADH, calculated for MDA-MB-231 and MDA-MB-231/R cells.

Figure S4. Workflow illustrating the treatment protocol of triple-negative breast cancer cDDP-sensitive (MDA-MB-231) and -resistant (MDA-MB-231/R) cell lines.

Table S1. Statistically significant metabolite variations observed in MDA-MB-231 cDDP-sensitive (S) and -resistant (R) cells treated with cDDP, compared to controls.

Table S2. Statistically significant metabolite variations observed in MDA-MB-231/R (R) cells directly compared to MDA-MB-231 (S) cells, considering controls (left), cDDP-treated (middle) and Pd_2Spm-treated (right) groups, at 48 h only.

Table S3. Statistically significant metabolite variations observed in MDA-MB-231 cDDP-sensitive (S) and -resistant (R) cells treated with Pd_2Spm, compared to controls.

Figure S1. Average 500 MHz ^{1}H-NMR spectra of aqueous extracts of cDDP-sensitive MDA-MB-231 cells (a) untreated and treated with (b) cDDP and (c) Pd_2Spm, after 48 h. * Cut-off of water suppression region (δ 4.4–5.4), not considered in the multivariate analysis. The arrows identify metabolic variations found with visual inspection of spectra of treated-groups in relation to controls. Abbreviations: 3-letter code for amino acids; Ac., acetate; Ado, adenosine; ADP, adenosine diphosphate; AMP, adenosine monophosphate; ATP, adenosine triphosphate; BCAAs, branched-chain amino acids (Ile, Leu and Val); Cho, choline; Cr, creatine; EtOH, ethanol; Fum., fumarate; GABA, γ-aminobutyrate; Glyc., glycerol; GPC, glycerophosphocholine; GSH, glutathione (reduced); HX, hypoxanthine; Ino, inosine; *m*-Ino, *myo*-Inositol; Lac., lactate; NAD^+, nicotinamide adenine dinucleotide (oxidized); PA, pantothenate; PC, phosphocholine; Pseudourd., pseudouridine; Tau, taurine; UDP-Glc/GlcA, uridine diphosphate-glucose/glucuronate; UDP-GlcNAc, uridine diphosphate *N*-acetylglucosamine; Urc., uracil.

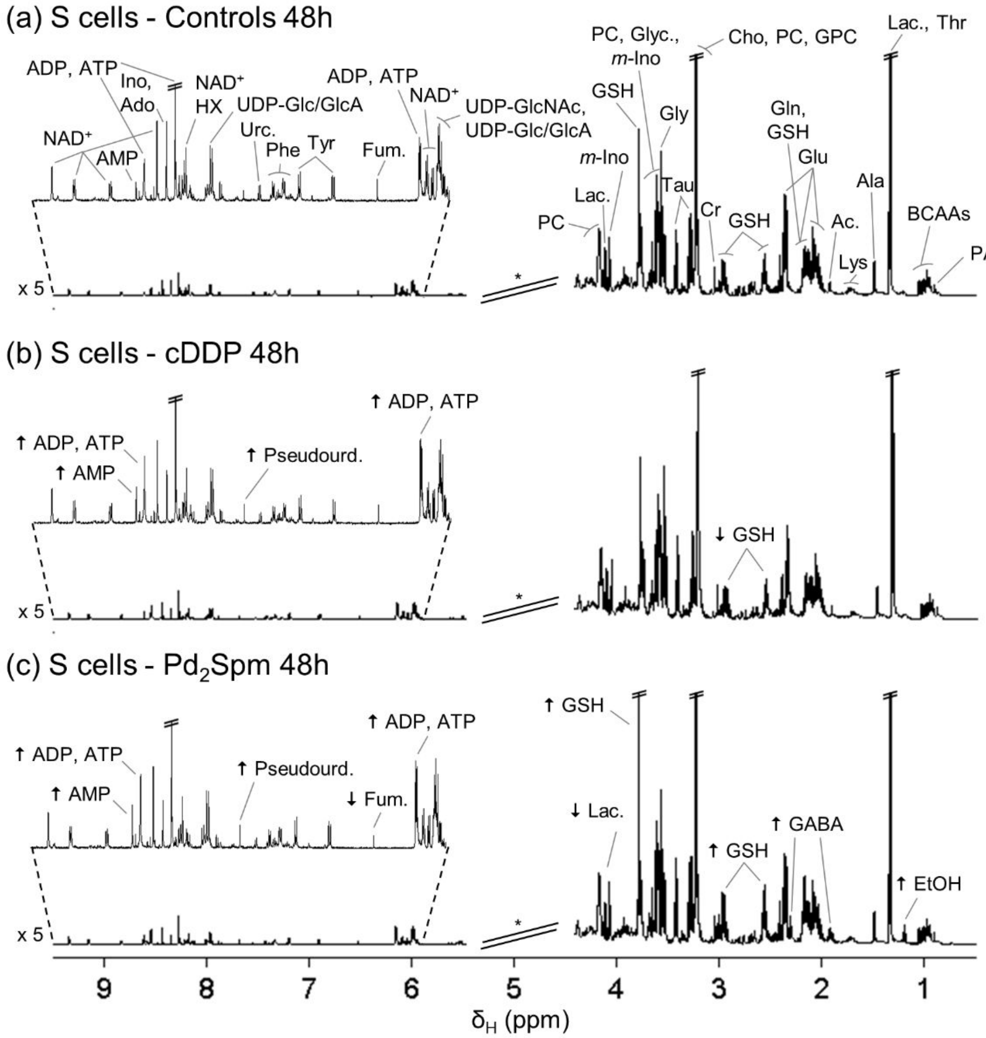

Figure S2. Bar charts illustrating time-course variations of (a) amino acids, (b) nucleotides and (c) other compounds significantly changed in MDA-MB-231 (S, striped bars) and MDA-MB-231/R (R, full bars) cells treated either with PBS (controls, black), cDDP (blue) or Pd_2Spm (red). Values are expressed as mean of normalized area of integrated peak ± SEM. Abbreviations: DMA, dimethylamine; IMP, inosine monophosphate; UMP, uridine monophosphate; other abbreviations as defined in Figure S1. Significant differences between treated-groups and controls in S and R cell lines at each time-point of treatment: * p-value < 0.05; ** p-value < 0.01; *** p-value < 0.001; **** p-value < 0.0001.

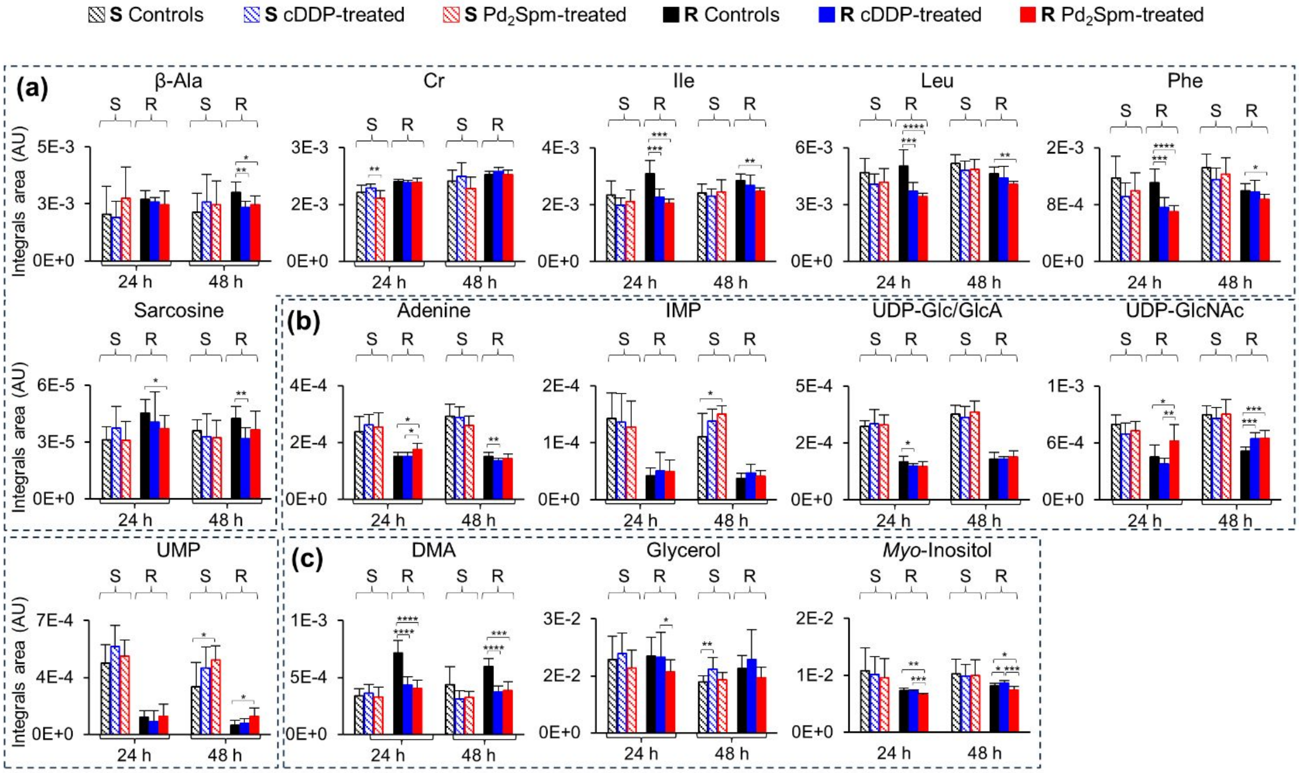

Figure S3. Bar charts illustrating time-course variations of ratios (a) Gln/Glu, (b) Glu/GABA, (c) PC/Cho, (d) ADP/ATP, (e) AMP/ATP and (f) NAD^+/NADH, calculated for MDA-MB-231 (S, striped bars) and MDA-MB-231/R (R, full bars) cells treated either with PBS (controls, black), cDDP (blue) or Pd_2Spm (red). Values are expressed as ratios mean of normalized area of integrated peak ± SEM. Abbreviations: NADH, nicotinamide adenine dinucleotide (reduced); other abbreviations as defined in Figure S1. Significant differences between treated-groups and controls in S and R cell lines at each time-point of treatment: * *p*-value < 0.05; ** *p*-value < 0.01; *** *p*-value < 0.001; **** *p*-value < 0.0001.

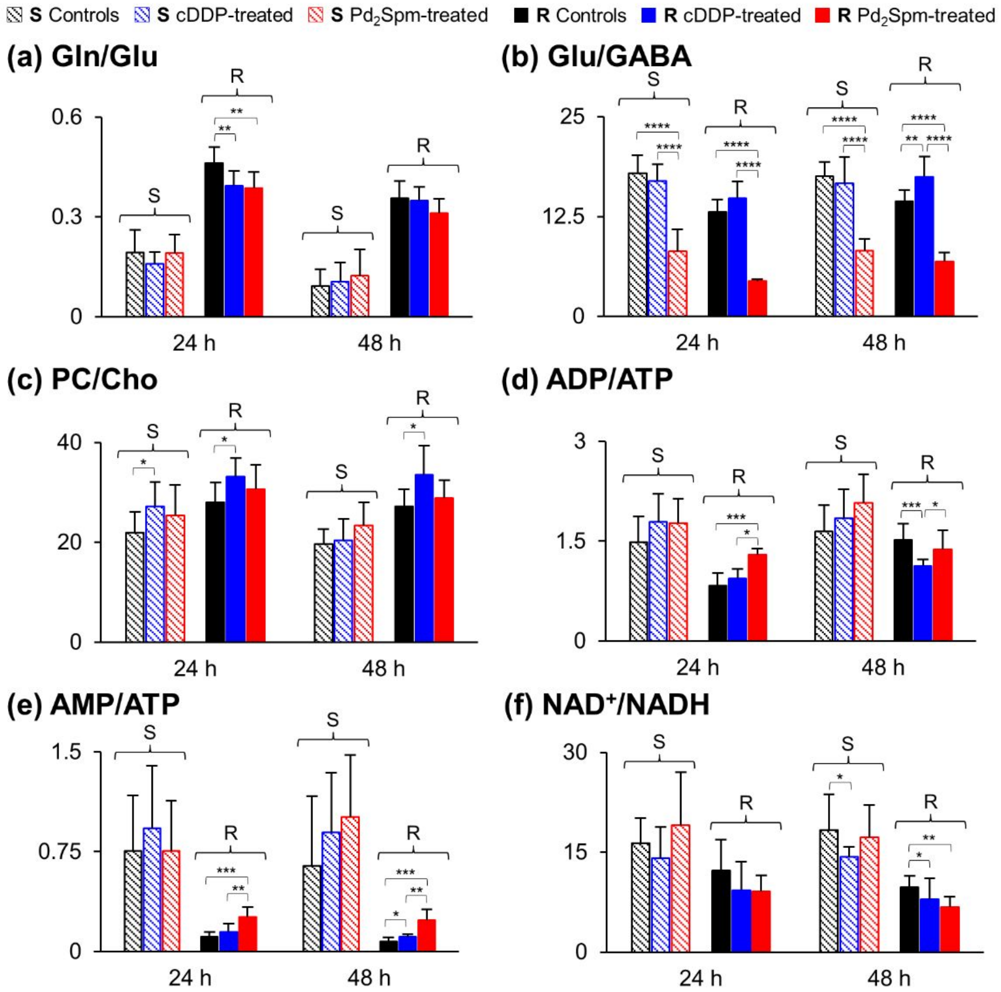

Figure S4. Workflow illustrating the treatment protocol of triple-negative breast cancer cDDP-sensitive (MDA-MB-231) and -resistant (MDA-MB-231/R) cell lines with vehicle solution (phosphate-buffered saline, PBS) in controls, and metal-drugs, cDDP (1.0 µM) or Pd_2Spm (7.9 µM); n = 3 independent assays (triplicates/ treatment and time-point).

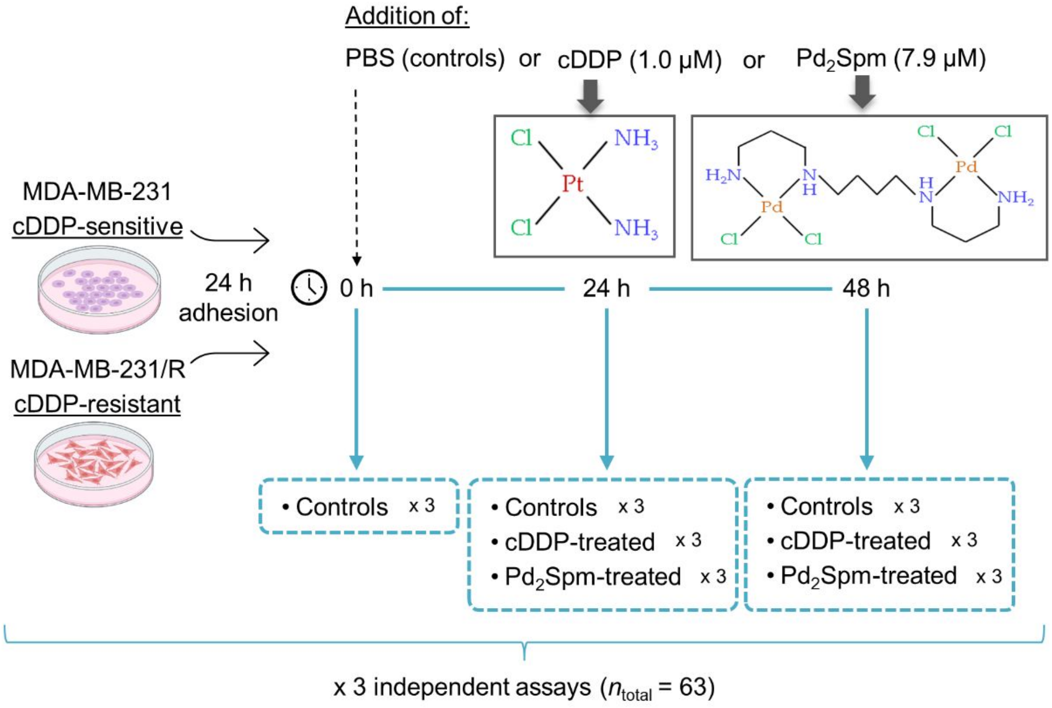

Table S1. Statistically significant (|ES| > ES Error and *p*-value < 0.05) metabolite variations observed in MDA-MB-231 cDDP-sensitive (S) and -resistant (R) cell lines treated with cDDP, compared to controls, after 24 and 48 h of treatment.

			**S cells**: cDDP *vs.* Ctr				**R cells**: cDDP *vs.* Ctr			
	**Metabolite**	**δ_H (multiplicity)**	24 h		48 h		24 h		48 h	
			ES ± Error	*p*-value	ES ± Error	*p*-value	ES ± Error	*p*-value	ES ± Error	*p*-value
Amino acids and derivatives	β-Ala	3.18 (t)	—	—	—	—	—	—	-1.7 ± 1.1	1.9E-3 [a]
	Ala	1.48 (d)	—	—	—	—	-2.0 ± 1.1	1.2E-3 [a]	—	—
	Asp	2.82 (dd)	—	—	-1.7 ± 1.1	2.8E-3 [a]	—	—	—	—
	Gln	2.45 (m)	—	—	—	—	-1.7 ± 1.1	3.4E-3 [a]	—	—
	Glu	2.36 (m)	—	—	-1.5 ± 1.1	5.8E-3 [a]	—	—	—	—
	Gly	3.55 (s)	—	—	—	—	-1.6 ± 1.1	8.5E-3 [a]	—	—
	GSH	2.96 (m)	—	—	-1.3 ± 1.0	1.5E-2	—	—	—	—
	Ile	0.94 (t)	—	—	—	—	-2.1 ± 1.1	6.9E-4 [a]	—	—
	Leu	0.96 (t)	—	—	—	—	-1.9 ± 1.1	2.9E-4 [a]	—	—
	Lys	1.73 (m)	—	—	-1.2 ± 1.0	2.3E-2	-2.1 ± 1.2	1.2E-3 [a]	-1.4 ± 1.0	1.1E-2 [a]
	Met	2.64 (t)	1.3 ± 1.0	1.4E-2	—	—	-2.5 ± 1.2	7.8E-5 [a]	-1.2 ± 1.0	2.1E-2
	NAA	2.02 (s)	—	—	-1.4 ± 1.0	3.2E-2	-2.2 ± 1.2	7.6E-4 [a]	-1.2 ± 1.0	1.8E-2
	PCr	3.05 (s)	2.4 ± 1.2	1.2E-4 [a]	-1.2 ± 1.0	1.9E-2	—	—	-1.3 ± 1.0	1.7E-2
	Phe	7.33 (m)	—	—	—	—	-2.4 ± 1.2	2.0E-4 [a]	—	—
	Pro	1.98 (m)	—	—	—	—	-1.7 ± 1.1	2.4E-3 [a]	-1.2 ± 1.0	2.4E-2
	Sarcosine	2.76 (s)	—	—	—	—	—	—	-1.7 ± 1.1	2.1E-3 [a]
	Tau	3.43 (t)	—	—	1.1 ± 0.9	3.2E-2	—	—	—	—
	Tyr	7.20 (d)	—	—	—	—	-2.1 ± 1.2	4.1E-5 [a]	—	—
	Val	1.05 (d)	—	—	—	—	-2.2 ± 1.2	4.1E-5 [a]	—	—
Cho cpd.	GPC	3.23 (s)	—	—	—	—	1.3 ± 1.0	1.6E-2	1.6 ± 1.1	5.8E-3 [a]
	PC	3.22 (s)	—	—	—	—	2.1 ± 1.2	9.7E-4 [a]	—	—
Nucleotides and derivatives	Adenine	8.19 (s)	—	—	—	—	—	—	-1.2 ± 1.0	2.5E-2
	Ado	8.27 (s)	1.0 ± 0.9	4.9E-2	—	—	—	—	—	—
	ADP	8.54 (s)	1.2 ± 1.0	1.1E-2 [a]	2.0 ± 1.1	5.4E-4 [a]	2.1 ± 1.2	3.6E-4 [a]	—	—
	AMP	8.61 (s)	—	—	1.6 ± 1.1	5.6E-3 [a]	1.1 ± 0.9	2.4E-2	2.4 ± 1.2	1.5E-4 [a]
	ATP	8.55 (s)	—	—	—	—	—	—	1.8 ± 1.1	2.8E-3 [a]
	HX	8.20 (s)	—	—	-2.2 ± 1.2	2.5E-4 [a]	—	—	—	—
	Ino, Ado	8.35 (s)	—	—	-2.2 ± 1.2	2.3E-4 [a]	—	—	—	—
	NAD$^+$	8.43 (s)	1.1 ± 0.9	3.2E-2	—	—	—	—	—	—
	NADH	8.48 (s)	—	—	—	—	—	—	1.2 ± 1.0	3.1E-2
	Pseudouridine	7.68 (s)	1.7 ± 1.1	2.4E-3 [a]	1.6 ± 1.1	3.6E-3 [a]	1.5 ± 1.1	7.7E-3 [a]	—	—
	UDP	8.01 (d)	—	—	1.7 ± 1.1	3.1E-3 [a]	—	—	1.9 ± 1.1	1.3E-3 [a]
	UDP-Glc/GlcA	7.95 (d)	—	—	—	—	-1.1 ± 0.9	4.4E-2	—	—
	UDP-GlcNAc	5.52 (dd)	—	—	—	—	—	—	2.3 ± 1.2	2.2E-4 [a]
	Uracil	5.81 (d)	—	—	-2.7 ± 1.3	4.1E-5 [a]	—	—	2.5 ± 1.2	7.8E-5 [a]
	Uridine	7.88 (d)	—	—	-1.6 ± 1.1	4.2E-3 [a]	—	—	—	—
Organic acids	Acetate	1.92 (s)	—	—	—	—	—	—	-1.5 ± 1.0	8.6E-3 [a]
	Citrate	2.70 (d)	1.2 ± 1.0	1.4E-2	-1.1 ± 0.9	3.9E-2	-1.6 ± 1.1	4.1E-3 [a]	-1.5 ± 1.0	7.8E-3 [a]
	Fumarate	6.52 (s)	—	—	-1.4 ± 1.0	9.3E-3 [a]	—	—	—	—
	Lactate	4.10 (q)	-1.3 ± 1.0	1.3E-2 [a]	—	—	—	—	-1.0 ± 0.9	4.8E-2
	Malate	2.67 (dd)	1.0 ± 0.9	4.9E-2	-1.4 ± 1.0	1.1E-2 [a]	-1.4 ± 1.0	1.2E-2 [a]	-1.2 ± 1.0	2.9E-2
	Pantothenate	0.90 (s)	—	—	—	—	1.7 ± 1.1	2.5E-3 [a]	—	—
	Succinate	2.41 (s)	—	—	—	—	-1.7 ± 1.1	3.0E-3 [a]	—	—
Other cpd.	Glycerol	3.65 (dd)	—	—	1.4 ± 1.0	9.6E-3 [a]	—	—	—	—
	Myo-Inositol	4.06 (t)	—	—	—	—	—	—	1.3 ± 1.0	1.6E-2

[a] Metabolic variation statistically significant after False Discovery Rate (FDR) correction (*p*-value > 0.05). Abbreviations: 3-letter code for amino acids; NAA, *N*-Acetyl-aspartate; PCr, phosphocreatine; UDP, uridine diphosphate; other abbreviations as defined in Figures S1 and S2.

Table S2. Statistically significant metabolite variations (|ES| > ES Error and *p*-value < 0.05, with ES Error ≤ ES) observed in MDA-MB-231/R (R) cells directly compared to MDA-MB-231 (S) cells, considering controls (left), cDDP-treated (middle) and Pd_2Spm-treated (right) groups, at 48 h only.

Comparison between R *vs.* S cell lines (48 h only)

	**Metabolite**	**δ_H (multiplicity)**	No treatment 48 h ES ± Error	No treatment 48 h *p*-value	cDDP 48 h ES ± Error	cDDP 48 h *p*-value	Pd_2Spm 48 h ES ± Error	Pd_2Spm 48 h *p*-value
Amino acids and derivatives	β-Ala [a]	3.18 (t)	1.3 ± 1.0	1.9E-2	—	—	—	—
	Ala [b]	1.48 (d)	-1.7 ± 1.1	3.2E-3	-1.1 ± 1.0	5.0E-2	-1.8 ± 1.1	2.0E-3
	Asp [b]	2.82 (dd)	—	—	2.0 ± 1.1	1.1E-3	2.0 ± 1.1	7.5E-4
	Cr [c]	3.04 (s)	—	—	—	—	1.6 ± 1.1	6.7E-3
	Gln [c]	2.45 (m)	3.9 ± 1.6	4.1E-5	3.3 ± 1.4	2.9E-4	—	—
	Glu [b]	2.36 (m)	-4.5 ± 1.8	1.9E-7	-3.0 ± 1.4	2.0E-5	-2.5 ± 1.2	3.1E-4
	Gly [c]	3.55 (s)	1.3 ± 1.0	1.4E-2	—	—	-1.3 ± 1.0	1.9E-2
	GSH [c]	2.96 (m)	3.2 ± 1.4	1.3E-5	7.7 ± 2.7	2.5E-10	2.6 ± 1.3	1.6E-4
	Ile [c]	0.94 (t)	1.6 ± 1.1	4.3E-3	1.2 ± 1.0	2.8E-2	—	—
	Leu [c]	0.96 (t)	-1.3 ± 1.0	1.9E-2	—	—	-2.1 ± 1.1	1.6E-3
	Lys [b]	1.73 (m)	-3.7 ± 1.5	2.0E-5	-3.2 ± 1.4	1.9E-5	-4.9 ± 1.8	5.3E-8
	Met [b]	2.64 (t)	6.1 ± 2.2	8.1E-10	3.4 ± 1.4	2.6E-6	4.0 ± 1.6	3.2E-7
	NAA [c]	2.02 (s)	—	—	-1.8 ± 1.1	5.6E-3	—	—
	PCr [b]	3.05 (s)	2.5 ± 1.2	7.7E-5	3.1 ± 1.4	1.5E-5	3.4 ± 1.4	1.3E-5
	Phe [b]	7.33 (m)	-2.7 ± 1.3	4.1E-5	-1.2 ± 1.0	2.0E-2	-2.5 ± 1.2	2.6E-4
	Pro [b]	1.98 (m)	-2.4 ± 1.2	7.7E-4	-3.3 ± 1.4	3.6E-5	-3.0 ± 1.3	1.8E-5
	Tau [b]	3.43 (t)	4.1 ± 1.6	5.0E-6	2.6 ± 1.3	4.1E-5	3.0 ± 1.3	4.1E-5
	Tyr [b]	7.20 (d)	-2.1 ± 1.2	8.8E-4	-1.1 ± 1.0	4.0E-2 †	-2.1 ± 1.2	1.4E-3
Cho cpd.	Cho [c]	3.20 (s)	-1.6 ± 1.1	5.9E-3	-2.7 ± 1.3	1.2E-4	—	—
	GPC [c]	3.23 (s)	—	—	-1.4 ± 1.0	1.6E-2	—	—
	PC [c]	3.21 (s)	—	—	—	—	1.3 ± 1.0	2.0E-2
Nucleotides and derivatives	Adenine [c]	8.19 (s)	-4.5 ± 1.7	2.6E-6	-6.0 ± 2.2	4.0E-7	-4.4 ± 1.7	1.1E-6
	Ado [b]	8.27 (s)	-4.4 ± 1.7	1.0E-5	-4.7 ± 1.8	5.6E-6	-3.1 ± 1.4	4.7E-5
	ADP [b]	8.54 (s)	—	—	-2.1 ± 1.2	8.0E-4	-1.9 ± 1.1	3.4E-3
	AMP [b]	8.61 (s)	-2.0 ± 1.1	4.1E-5	-4.0 ± 1.6	2.5E-5	-3.7 ± 1.5	2.6E-5
	HX [b]	8.20 (s)	-6.5 ± 2.3	3.2E-9	-4.7 ± 1.8	1.1E-6	-2.0 ± 1.1	1.5E-3
	IMP [c]	8.58 (s)	-2.5 ± 1.2	5.2E-4	-4.8 ± 1.8	4.6E-8	-9.0 ± 3.1	2.1E-11
	Ino, Ado [c]	8.35 (s)	-6.4 ± 2.3	2.8E-8	-3.3 ± 1.4	6.8E-5	—	—
	NAD^+ [b]	8.43 (s)	-5.9 ± 2.1	8.2E-7	-6.5 ± 2.3	1.0E-7	-4.5 ± 1.7	7.2E-6
	NADH [c]	8.48 (s)	—	—	—	—	1.1 ± 1.0	3.5E-2
	Pseudouridine [b]	7.68 (s)	—	—	-1.4 ± 1.0	1.1E-2	-1.2 ± 1.0	3.1E-2
	UDP [b]	8.10 (d)	-2.0 ± 1.1	9.1E-4	-2.4 ± 1.2	9.8E-5	-1.2 ± 1.0	1.9E-2
	UDP-Glc/GlcA [b]	7.95 (d)	-6.0 ± 2.2	2.5E-9	-5.2 ± 1.9	1.7E-6	-4.9 ± 1.8	1.7E-7
	UDP-GlcNAc [b]	5.52 (dd)	-5.0 ± 1.9	3.0E-7	-2.3 ± 1.2	3.2E-4	-2.0 ± 1.1	1.1E-3
	UMP [b]	8.11 (s)	-2.2 ± 1.2	1.4E-3	-3.6 ± 1.5	4.6E-5	-4.9 ± 1.8	1.3E-7
	Uracil [a]	5.81 (d)	-6.3 ± 2.3	4.1E-5	—	—	—	—
	Uridine [b]	7.88 (d)	-6.1 ± 2.2	2.0E-9	-2.0 ± 1.1	2.3E-3	—	—
Organic acids	Acetate [c]	1.92 (s)	-2.8 ± 1.3	5.1E-5	-6.1 ± 2.2	1.4E-9	-1.7 ± 1.1	5.0E-3
	Citrate [c]	2.70 (d)	-1.5 ± 1.0	6.8E-3	-1.3 ± 1.0	2.3E-2	—	—
	Fumarate [c]	6.52 (s)	-3.2 ± 1.4	3.2E-5	-1.5 ± 1.1	6.3E-3	—	—
	Lactate [b]	4.10 (q)	-2.3 ± 1.2	2.8E-4	-3.1 ± 1.4	2.1E-5	-4.0 ± 1.6	5.2E-7
	Pantothenate [c]	0.90 (s)	-1.1 ± 1.0	4.9E-2 †	—	—	1.1 ± 1.0	4.0E-2 †
	Succinate [b]	2.41 (s)	-1.4 ± 1.0	2.8E-3	-2.3 ± 1.2	8.2E-5	-1.7 ± 1.1	6.6E-3
Other cpd.	Glycerol [a]	3.65 (dd)	1.3 ± 1.0	1.5E-2	—	—	—	—
	Myo-Inositol [a]	4.06 (t)	-1.1 ± 1.0	4.7E-2 †	—	—	—	—

All variations remained significant after False Discovery Rate (FDR) correction (*p*-value > 0.05), except for those labelled with †. Differences between R *vs.* S, observed [a] only in controls, [b] similar (in direction and magnitude) between cDDP and Pd_2Spm, and [c] different between cDDP and Pd_2Spm (these are also shaded, for the sake of clarity). Abbreviations as defined in Figures S1 and S2 as well as Table S1.

Table S3. Statistically significant (|ES| > ES Error and *p*-value < 0.05) metabolite variations observed in MDA-MB-231 cDDP-sensitive (S) and -resistant (R) cell lines treated with Pd_2Spm, compared to controls, after 24 and 48 h of treatment.

			**S cells**: Pd_2Spm *vs.* Ctr				**R cells**: Pd_2Spm *vs.* Ctr			
	**Metabolite**	**δ_H (multiplicity)**	24 h		48 h		24 h		48 h	
			ES ± Error	*p*-value	ES ± Error	*p*-value	ES ± Error	*p*-value	ES ± Error	*p*-value
Amino acids and derivatives	β-Ala	3.18 (t)	—	—	—	—	—	—	-1.3 ± 1.0	3.2E-2
	Ala	1.48 (d)	—	—	-1.0 ± 0.9	4.8E-2	-3.8 ± 1.6	9.8E-6 [a]	-2.3 ± 1.2	2.5E-4 [a]
	Asp	2.82 (dd)	-1.2 ± 1.0	2.3E-2 [a]	-1.7 ± 1.1	2.7E-3 [a]	—	—	—	—
	GABA	2.30 (t)	2.4 ± 1.2	2.9E-4 [a]	7.0 ± 2.5	4.1E-5 [a]	13.1 ± 4.4	1.2E-12 [a]	5.6 ± 2.0	4.1E-5 [a]
	Gln	2.45 (m)	—	—	—	—	-2.7 ± 1.3	8.6E-5 [a]	-2.4 ± 1.2	1.6E-4 [a]
	Glu	2.36 (m)	-6.3 ± 2.3	6.6E-10 [a]	-1.9 ± 1.1	9.6E-4 [a]	-3.4 ± 1.4	1.3E-5 [a]	-2.0 ± 1.1	5.8E-4 [a]
	Gly	3.55 (s)	-1.7 ± 1.1	2.8E-3 [a]	—	—	-7.7 ± 2.7	1.2E-9 [a]	-2.8 ± 1.3	4.9E-4 [a]
	GSH	2.96 (m)	6.6 ± 2.4	2.0E-9 [a]	3.8 ± 1.6	4.1E-5 [a]	2.1 ± 1.2	1.3E-3 [a]	3.2 ± 1.4	4.0E-6 [a]
	Ile	0.94 (t)	—	—	—	—	-2.9 ± 1.3	1.3E-4 [a]	-2.1 ± 1.1	1.0E-3 [a]
	Leu	0.96 (t)	—	—	—	—	-2.6 ± 1.3	4.1E-5 [a]	-2.0 ± 1.1	1.5E-3 [a]
	Lys	1.73 (m)	—	—	-1.6 ± 1.1	4.6E-3 [a]	-3.2 ± 1.4	7.9E-5 [a]	-2.8 ± 1.3	2.9E-5 [a]
	Met	2.64 (t)	1.0 ± 0.9	4.9E-2	-	-	—	—	—	—
	NAA	2.02 (s)	-2.7 ± 1.3	3.1E-5 [a]	-3.5 ± 1.5	1.3E-5 [a]	-3.3 ± 1.4	6.8E-5 [a]	-2.0 ± 1.1	4.9E-4 [a]
	PCr	3.05 (s)	1.2 ± 1.0	7.8E-3 [a]	-1.4 ± 1.0	1.2E-2 [a]	—	—	-2.0 ± 1.1	1.6E-3 [a]
	Phe	7.33 (m)	—	—	—	—	-2.9 ± 1.3	4.6E-5 [a]	-1.2 ± 1.0	2.6E-2 [a]
	Pro	1.98 (m)	-1.3 ± 1.0	1.7E-2 [a]	-1.1 ± 0.9	3.2E-2	-3.0 ± 1.4	1.5E-5 [a]	-2.6 ± 1.3	1.6E-4 [a]
	Sarcosine	2.76 (s)	—	—	—	—	-1.2 ± 1.0	2.5E-2 [a]	—	—
	Tau	3.43 (t)	—	—	—	—	1.4 ± 1.0	1.1E-2 [a]	—	—
	Tyr	7.20 (d)	—	—	—	—	-2.8 ± 1.3	4.1E-5 [a]	-1.4 ± 1.0	8.3E-3 [a]
	Val	1.05 (d)	—	—	—	—	-2.9 ± 1.3	4.1E-5 [a]	-2.1 ± 1.2	1.2E-3 [a]
Cho cpd.	Cho	3.20 (s)	—	—	—	—	1.2 ± 1.0	2.0E-2 [a]	-	-
	GPC	3.23 (s)	—	—	—	—	3.5 ± 1.5	1.5E-6 [a]	2.0 ± 1.1	8.7E-4 [a]
	PC	3.22 (s)	—	—	—	—	2.1 ± 1.2	1.3E-3 [a]	2.4 ± 1.2	2.5E-4 [a]
Nucleotides and derivatives	Adenine	8.19 (s)	—	—	—	—	1.3 ± 1.0	1.3E-2 [a]	—	—
	ADP	8.54 (s)	—	—	1.9 ± 1.1	1.1E-3 [a]	3.3 ± 1.4	4.5E-6 [a]	—	—
	AMP	8.61 (s)	—	—	1.8 ± 1.1	4.0E-3 [a]	2.5 ± 1.2	2.6E-4 [a]	3.4 ± 1.5	1.0E-5 [a]
	ATP	8.55 (s)	—	—	—	—	-1.4 ± 1.0	1.5E-2 [a]	—	—
	HX	8.20 (s)	—	—	-3.1 ± 1.4	8.9E-6 [a]	—	—	—	—
	IMP	8.58 (s)	—	—	1.3 ± 1.0	2.2E-2 [a]	—	—	—	—
	Ino, Ado	8.35 (s)	-2.1 ± 1.2	4.3E-4 [a]	-2.1 ± 1.2	6.5E-4 [a]	—	—	1.6 ± 1.1	4.3E-3 [a]
	NAD^+	8.43 (s)	—	—	—	—	1.5 ± 1.1	7.1E-3 [a]	1.2 ± 1.0	1.9E-2 [a]
	NADH	8.48 (s)	—	—	—	—	1.1 ± 0.9	4.0E-2	2.6 ± 1.3	8.0E-5
	Pseudouridine	7.68 (s)	—	—	1.2 ± 1.0	7.8E-3 [a]	—	—	—	—
	UDP	8.01 (d)	—	—	2.6 ± 1.2	5.8E-5 [a]	1.5 ± 1.1	5.1E-3 [a]	4.6 ± 1.8	7.4E-8 [a]
	UDP-GlcNAc	5.52 (dd)	—	—	—	—	1.1 ± 1.0	2.9E-2	2.1 ± 1.2	7.3E-4 [a]
	UMP	8.11 (s)	—	—	1.3 ± 1.0	1.4E-2 [a]	—	—	1.3 ± 1.0	1.7E-2 [a]
	Uracil	5.81 (d)	—	—	-2.1 ± 1.2	4.9E-4 [a]	—	—	1.3 ± 1.0	1.7E-2 [a]
	Uridine	7.88 (d)	—	—	-1.7 ± 1.1	4.0E-3 [a]	—	—	1.6 ± 1.1	4.1E-3 [a]
Organic acids	Acetate	1.92 (s)	1.4 ± 1.0	1.1E-2 [a]	1.3 ± 1.0	1.4E-2 [a]	5.6 ± 2.1	4.7E-8 [a]	4.2 ± 1.6	1.6E-7 [a]
	Citrate	2.70 (d)	—	—	-1.4 ± 1.0	1.1E-2 [a]	-2.0 ± 1.1	1.1E-3 [a]	-1.5 ± 1.0	7.3E-3 [a]
	Fumarate	6.52 (s)	-3.0 ± 1.3	1.2E-5 [a]	-2.7 ± 1.3	2.8E-5 [a]	—	—	-1.0 ± 0.9	4.8E-2
	Lactate	4.10 (q)	-1.6 ± 1.1	3.1E-3 [a]	-3.1 ± 1.4	1.1E-5 [a]	-3.0 ± 1.4	1.4E-5 [a]	-2.6 ± 1.3	8.8E-5 [a]
	Malate	2.67 (dd)	—	—	-1.8 ± 1.1	2.1E-3 [a]	-1.3 ± 1.0	1.8E-2 [a]	-3.0 ± 1.3	8.2E-5 [a]
	Pantothenate	0.90 (s)	—	—	—	—	2.6 ± 1.3	4.6E-5 [a]	1.5 ± 1.0	6.5E-3 [a]
	Succinate	2.41 (s)	—	—	—	—	-2.0 ± 1.1	6.4E-4 [a]	—	—
Other cpd.	DMA	2.73 (s)	—	—	—	—	-3.4 ± 1.4	4.7E-6 [a]	-3.0 ± 1.3	4.9E-4 [a]
	Myo-Inositol	4.06 (t)	—	—	—	—	-2.0 ± 1.1	1.3E-3 [a]	-1.6 ± 1.1	1.1E-2 [a]

[a] Metabolic variation statistically significant after False Discovery Rate (FDR) correction (*p*-value > 0.05). Abbreviations as defined in Figures S1 and S2.
